# A new species of the genus *Theloderma* Tschudi, 1838 (Amphibia: Anura: Rhacophoridae) from Tay Nguyen Plateau, central Vietnam

**DOI:** 10.24272/j.issn.2095-8137.2018.018

**Published:** 2018-04-28

**Authors:** Nikolay A. Poyarkov, Ivan I. Kropachev, Svetlana S. Gogoleva, Nikolai L. Orlov

**Affiliations:** 1 Department of Vertebrate Zoology, Biological Faculty, Lomonosov Moscow State University, Moscow 119234, Russia; 2 Joint Russian-Vietnamese Tropical Research and Technological Center, Nghia Do, Cau Giay, Hanoi, Vietnam; 3 Tula Exotarium, Tula 300004, Russia; 4 A.N. Severtsov Institute of Ecology and Evolution, Russian Academy of Sciences, Moscow 119071, Russia; 5 Joint Russian-Vietnamese Tropical Research and Technological Center, South Branch, Ho Chi Minh City, Vietnam; 6 Zoological Museum of the Lomonosov Moscow State University, Moscow 125009, Russia; 7 Zoological Institute, Russian Academy of Sciences, St. Petersburg 199034, Russia

**Keywords:** *Theloderma auratum***sp. nov.**, mtDNA phylogeny, 12S rRNA, 16S rRNA, Kon Tum, Gia Lai, Endemism, Taxonomy

## Abstract

A new species of small tree frog from a primary montane tropical forest of central Vietnam, Tay Nguyen Plateau, is described based on morphological, molecular, and acoustic evidence. The Golden Bug-Eyed Frog, *Theloderma auratum* **sp. nov.**, is distinguishable from its congeners and other small rhacophorid species based on a combination of the following morphological attributes: (1) bony ridges on head absent; (2) smooth skin completely lacking calcified warts or asperities; (3) pointed elongated tapering snout; (4) vocal opening in males absent; (5) vomerine teeth absent; (6) males of small body size (SVL 21.8–26.4 mm); (7) head longer than wide; ED/SVL ratio 13%–15%; ESL/SVL ratio 16%–20%; (8) small tympanum (TD/EL ratio 50%–60%) with few tiny tubercles; (9) supratympanic fold absent; (10) ventral surfaces completely smooth; (11) webbing between fingers absent; (12) outer and inner metacarpal tubercles present, supernumerary metacarpal tubercle single, medial, oval in shape; (13) toes half-webbed: I 2–2¼ II 1½–2¾ III 2–3¼ IV 3–1½ V; (14) inner metatarsal tubercle present, oval; outer metatarsal tubercle absent; (15) iris bicolored; (16) dorsal surfaces golden-yellow with sparse golden-orange speckling or reticulations and few small dark-brown spots; (17) lateral sides of head and body with wide dark reddish-brown to black lateral stripes, clearly separated from lighter dorsal coloration by straight contrasting edge; (18) ventral surfaces of body, throat, and chest greyish-blue with indistinct brown confluent blotches; (19) upper eyelids with few (3–5) very small flat reddish superciliary tubercles; (20) limbs dorsally reddish-brown, ventrally brown with small bluish-white speckles. The new species is also distinct from all congeners in 12S rRNA to 16S rRNA mitochondrial DNA fragment sequences (uncorrected genetic distance *P*>8.9%). Advertisement call and tadpole morphology of the new species are described. Our molecular data showed *Theloderma auratum* **sp. nov.** to be a sister species of *Th. palliatum* from Langbian Plateau in southern Vietnam.

## INTRODUCTION

Rhacophoridae is a diverse family of largely arboreal tree frogs consisting of 414 currently recognized species in 18 genera (AmphibiaWeb, 2018; Che & Wang, 2016; Frost, 2018). Rhacophorids are distributed throughout Sub-Saharan Africa, China, Southern and Southeast Asia, Japan, Taiwan, the Philippines, and the Greater Sunda Islands (Frost, 2018). Species diagnostics and generic allocation within Rhacophoridae can be difficult, and molecular phylogenetic analyses are essential for correct taxonomic decisions (Jiang et al., 2016; Li et al., 2008, 2009; Orlov et al., 2012; Poyarkov et al., 2015; Rowley et al., 2011; Yu et al., 2009).

Frogs of the genus *Theloderma* Tschudi, 1838 are highly arboreal small- to large-sized rhacophorid frogs, which were traditionally grouped in one genus based on the presence of dorsal asperities or tuberculate dorsal skin (Liem, 1970; Rowley et al., 2011). However, recent studies have reported on several smooth species of rhacophorids that almost completely or totally lack dorsal asperities but are still assigned to *Theloderma* based on molecular data and reproductive biology (Nguyen et al., 2014; Orlov et al., 2012; Poyarkov et al., 2015). Thus, the taxonomy of *Theloderma* is in a current state of flux. Several studies have indicated a sister-clade relationship between *Nyctixalus* Boulenger, 1882 and *Theloderma*, with monophyly of the latter being poorly supported or not recovered (Dever et al., 2015; Dever, 2017; Rowley et al., 2011). Thus, Poyarkov et al. (2015) recognized *Nyctixalus* as a subgenus of *Theloderma* and assigned the *Th. stellatum* Taylor, 1962-*Th. horridum* (Boulenger, 1903) species group to the subgenus *Stelladerma* Poyarkov, Orlov, Moiseeva, Pawangkhanant, Ruangsuwan, Vassilieva, Galoyan, Nguyen & Gogoleva, 2015. Subsequent studies with larger taxon and gene sampling (Nguyen et al., 2015; Sivongxay et al., 2016) supported *Nyctixalus* as a sister clade with respect to *Theloderma* as well as monophyly of *Theloderma* and its subdivision into two highly divergent clades (corresponding to subgenera *Theloderma* and *Stelladerma sensu* Poyarkov et al., 2015). *Theloderma moloch* (Annandale, 1912), an enigmatic taxon whose phylogenetic position and assignment to *Theloderma* became uncertain due to sequences published by Li et al. (2009), was simultaneously rediscovered in Chinese and Indian regions of the Himalayas and shown to be a member of *Theloderma* s. str. (Hou et al., 2017; Lalronunga & Lalrinchhana, 2017; Li et al., 2016). In addition, Jiang et al. (2016) and Biju et al. (2016) concurrently demonstrated that sequences previously reported as “*Th. moloch*” by Li et al. (2009) actually corresponded to a distinct lineage of Rhacophoridae, representing new genus *Nasutixalus* Jiang, Yan, Wang & Che, 2016 (Jiang et al., 2016). The taxonomic position of *Th. andersoni* (Ahl, 1927) is also relatively unclear, with Hou et al. (2017) suggesting that this species might be a member of the genus *Raorchestes* Biju, Shouche, Dubois, Dutta & Bossuyt, 2010. Taking these reassignments and new species descriptions into consideration, a total of 25 species of *Theloderma* are recognized and distributed throughout Southeast Asia, from Assam in northeastern India to Myanmar, southern China and Indochina, the Malay Peninsula, and Sumatra and Borneo of the Greater Sunda Islands. New species in the genus continue to be discovered, with 13 described in the last 15 years alone (Frost, 2018).

During recent fieldwork in the montane forests of Tay Nguyen Plateau in central Vietnam, we encountered a small-sized rhacophorid species with a smooth dorsum. This tree frog was identified morphologically as a probable member of the genus *Theloderma* based on the presence of an intercalary cartilage between the terminal and penultimate phalanges of digits, a distinct tympanum, tips of the digits expanded into large disks bearing circummarginal grooves, absence of vomerine teeth, horizontal pupils, rounded canthus rostralis, terminal phalanx with a Y-shaped distal end, and the skin of the head not co-ossified to the skull (Liem, 1970; McLeod & Ahmad, 2007; Rowley et al., 2011). This species of frog was earlier reported by Orlov & Ananjeva (2007) and Orlov et al. (2012) as *Theloderma laeve* (Smith, 1924), a taxon described from the Langbian Plateau of southern Vietnam and originally assigned to the genus *Philautus* due to the presence of smooth skin on the dorsum and the lack of warts, tubercles, and asperities (Smith, 1924). Following examination of the holotype of *Philautus laevis* Smith, 1924, Poyarkov et al. (2015) demonstrated it to be conspecific to *Theloderma bambusicolum* Orlov, Poyarkov, Vassilieva, Ananjeva, Nguyen, Nguyen & Geissler, 2012 and confirmed its assignment to the genus *Theloderma* as *Theloderma laeve* (Smith, 1924). Consequently, the taxon previously identified as *Th. laeve* by Orlov et al. (2012) is not assigned to any currently recognized species of *Theloderma*. Close examination showed that this taxon can be easily distinguished from other known members of the genus *Theloderma* by a combination of several adult morphological characteristics. Molecular phylogenetic analysis of a 2 518-bp mtDNA fragment further indicated that this taxon is nested within the genus *Theloderma* with high support values and represents a sister species to *Th. palliatum* Rowley, Le, Hoang, Dau & Cao, 2011. In the present paper we discuss the phylogenetic position and taxonomic affiliation of this frog and describe it as a new species.

## MATERIALS AND METHODS

### Sample collection

**Figure 1:**
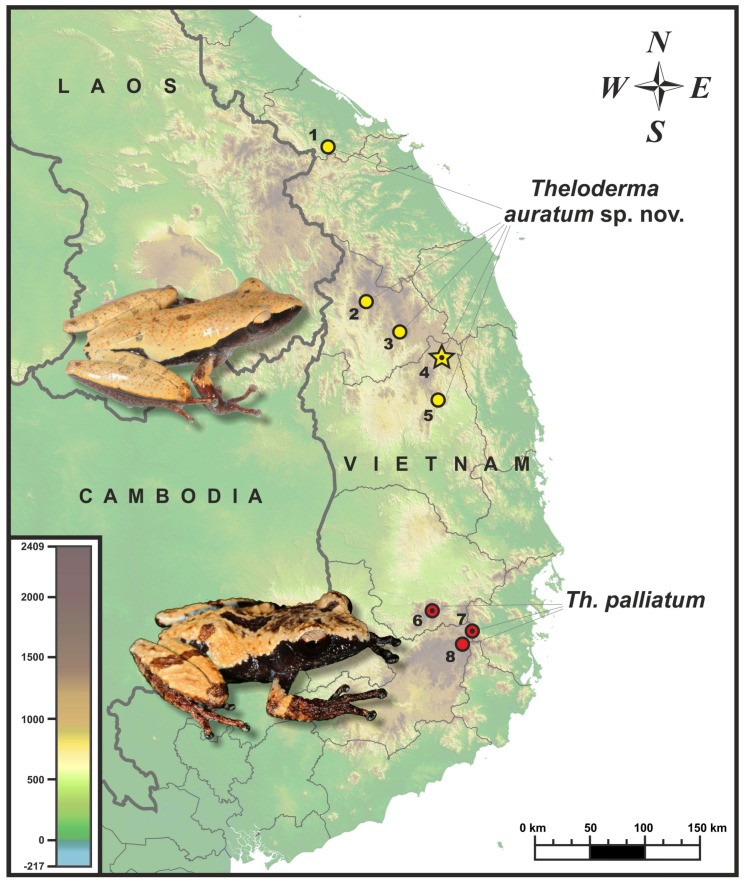
Known distribution of *Theloderma auratum* sp. nov. and *Th. palliatum* in Vietnam

Fieldwork was carried out in central Vietnam in May 2016 and May 2017. Specimens of *Theloderma* sp. were collected by hand in the montane evergreen tropical forests of Kon Ka Kinh National Park and Kon Chu Rang Nature Reserve, both in Gia Lai Province; and in Thac Nham Forest and Kon Plinh Forest, Mang Canh District, Kon Tum Province; Tay Nguyen Plateau, central Vietnam (Figure 1). Geographic coordinates and altitude were obtained using a Garmin GPSMAP 60CSx GPS receiver (USA) and recorded in datum WGS 84. Specimens were euthanized by 20% benzocaine and tissue samples (femoral muscles) for genetic analysis were collected and stored in 96% ethanol prior to specimen preservation. Specimens were subsequently preserved in 70% ethanol and deposited in the herpetological collection of the Zoological Museum of Moscow State University (ZMMU) in Moscow, Russia. Comparative materials examined are stored in the herpetological collections of ZMMU and the Zoological Institute R.A.S. (ZISP) in St. Petersburg, Russia. A list of examined specimens is given in Appendix I.

Star denotes type locality of new species; dot in circle denotes type locality for previously described species. Locality information: 1: A Luoi, Thua Thien – Hue Prov.; 2: Kon Plinh, Kon Tum Prov.; 3: Thac Nham, Mang Canh, Kon Tum Prov.; 4: Kon Chu Rang N.R., Gia Lai Prov. (type locality of *Theloderma auratum* **sp. nov.**); 5: Kon Ka Kinh N.P., Gia Lai Prov.; 6: Chu Yang Sin N.P., Dak Lak Prov. (type locality of *Theloderma chuyangsinense* Orlov, Poyarkov, Vassilieva, Ananjeva, Nguyen, Nguyen & Geissler, 2012); 7: Hon Giao Mt., Bidoup – Nui Ba N.P., Lam Dong Prov. (type locality of *Theloderma palliatum* Rowley, Le, Hoang, Dau & Cao, 2011); 8: Bidoup Mt., Bidoup – Nui Ba N.P., Lam Dong Prov. Photos by Nikolay A. Poyarkov.

### Morphological description

Specimens of *Theloderma* sp. were photographed in life and after preservation. Sex was determined by direct observation of calls and by examination of the nuptial pads in collected males. Measurements were taken using a digital caliper under a light dissecting microscope to the nearest 0.01 mm, subsequently rounded to 0.1 mm. Statistical analyses were performed with Statistica 8.0 (StatSoft, Inc., 2007).

### Adult morphology

Morphometrics follow Orlov et al. (2012), Milto et al. (2013), and Poyarkov et al. (2015) and include the following 30 measurements: (1) SVL (snout-vent length); (2) A-G (axilla to groin, distance from posterior base of forelimb at its emergence from body to anterior base of hind limb at its emergence from body); (3) HW (head width at greatest cranial width); (4) HL (head length from rear of lower jaw to tip of the snout); (5) HD (head depth, greatest transverse depth of head, taken beyond interorbital region); (6) UEW (upper eyelid width, greatest width of upper eyelids); (7) IOD (interorbital distance); (8) ED (horizontal diameter of eye); (9) TD (horizontal diameter of tympanum); (10) ESL (tip of snout-eye distance); (11) IND (internarial distance between nostrils); (12) END (eye to nostril distance from anterior corner of eye to nostril); (13) TED (tympanum-eye distance from anterior edge of tympanum to posterior corner of eye); (14) NS (distance from nostril to tip of snout); (15) FLL (length of forelimb from tip of disk of finger III to axilla); (16) LAL (forearm length, from elbow to base of outer palmar tubercle); (17) ML (hand length from tip of third digit to base of outer palmar tubercle); (18) FFL (first finger length); (19) TFL (third finger length); (20) FTD (maximal diameter of disk of finger III); (21) NPL (nuptial pad length, measured for males only); (22) MKTe (length of external metacarpal tubercle); (23) HLL (length of hindlimb from tip of disk of toe IV to groin); (24) FL (femur length); (25) TL (tibia length); (26) FOT (length of hindlimb from tip of disk of toe IV to posterior edge of tibia); (27) FTL (first toe length); (28) FFTL (fourth toe length); (29) HTD (diameter of fourth toe tip, greatest diameter of disk on fourth toe); (30) MTTi (length of internal metatarsal tubercle). Skin texture, dorsal coloration, ventral coloration, and presence of supratympanic folds, circummarginal grooves, dorsolateral folds, vomerine teeth, hind limb and forelimb webbing, and dorsal, lateral, and ventral coloration were recorded. Webbing formula follows Savage & Heyer (1997).

Data and adult morphological characters chosen for comparison with other *Theloderma* species and allied taxa were taken from the following sources: Ahl (1927, 1931); Anderson (1879); Annandale (1912); Bain et al. (2009); Boulenger (1882, 1886, 1893, 1903); Bourret (1937, 1942); Chan & Ahmad (2009); Chan-ard (2003); Das et al. (2013); Das (2007); Dever et al. (2015); Dever (2017); Fei et al. (1990, 2009, 2010); Hou et al. (2017); Inger (1954, 1966); Lalronunga & Lalrinchhana (2017); Li et al. (2011, 2016); Liem (1970); Liu & Hu (1962); Manthey & Grossmann (1997); Mathew & Sen. (2010); McLeod & Ahmad (2007); Nguyen et al. (2014); Orlov & Ananjeva (2007); Orlov & Ho (2005); Orlov et al. (2006, 2010, 2012); Orlov (2007); Peters (1871); Poyarkov et al. (2015); Rowley et al. (2011); Sivongxay et al. (2016); Smith (1924, 1926, 1930, 1931); Stuart & Heatwole (2004); Taylor (1920, 1962); Von Tschudi (1838); Wolf (1936); Zhao & Adler (1993).

### Larval morphology

Morphometric data and abbreviations for larval characters follow Altig & McDiarmid (1999), Altig (2007), Grosjean (2001), and Hendrix et al. (2007). Labial tooth row formulae (LTRF) follow Altig & McDiarmid (1999). Tadpoles were staged according to the tables of Gosner (1960). All measurements were taken on freshly preserved specimens. Tadpoles were euthanized with ethyl acetate and preserved in 70% ethanol. The following 17 measurements were taken: (1) BH (maximum body height); (2) BL (body length); (3) BW (maximum body width); (4) ED (maximum diameter of eye); (5) NP (naro-pupilar distance); (6) IND (internarial distance, measured between centers of narial apertures); (7) IOD (interorbital distance); (8) ODW (oral disk width); (9) RN (rostro-narial distance); (10) SS (distance from tip of snout to spiracle opening); (11) LF (maximum height of lower tail fin); (12) MTH (maximum tail height); (13) TAL (tail length); (14) TL (total length); (15) TMH (height of tail musculature at base); (16) TMW (width of tail musculature at base); (17) UF (maximum height of upper tail fin).

### DNA isolation, PCR, and sequencing

Total genomic DNA was extracted from the ethanol-preserved muscle tissues using standard phenol-chloroform extraction (Hillis et al., 1996). Total DNA concentration was estimated in 1 μL using a NanoDrop 2000 (Thermo Scientific, USA), and consequently adjusted to 100 ng DNA/μL.

We amplified the mtDNA fragment consisting of partial sequences of 12S rRNA, tRNA^val^, and 16S rRNA mtDNA genes. These markers were chosen due to their successful use in studies of Rhacophorid taxonomic diversity (Bain et al., 2009; Dever et al., 2015; Li et al., 2008, 2009, 2013; Meegaskumbura et al., 2015; Nguyen et al., 2014, 2015; Poyarkov et al., 2015; Rowley et al., 2011; Wilkinson & Drewes, 2000; Wilkinson et al., 2002; Yu et al., 2007, 2008; and references therein). PCR was performed in 20-μL reactions using 50 ng of genomic DNA, 10 nmol of each primer, 15 nmol of each dNTP, 50 nmol additional MgCl_2_, Taq PCR buffer (10 mmol/L Tris-HCl, pH 8.3, 50 mmol/L KCl, 1.1 mmol/L MgCl_2_, and 0.01% gelatin), and 1 U of Taq DNA polymerase. Primers used in PCR and sequencing were obtained from previous studies (Hedges, 1994; Li et al., 2008, 2009; Wilkinson et al., 2002) and are summarized in Table 1. The PCR conditions for amplification of the 12S rRNA and 16S rRNA gene fragments followed those described in Poyarkov et al. (2015).

**Table 1:** Primers used in this study

**Forward primers**	
**F0001**	5′-AGATACCCCACTATGCCTACCC-3′
**F0483**	5′-GAAGAGGCAAGTCGTAACATGG-3′
**F0937**	5′-TGGGATGATTTTCAAGTAG-3′
**F1624**	5′-GTATCAACGGCATCACGAGGG-3′
**16S-L-1**	5′-CTGACCGTGCAAAGGTAGCGTAATCACT-3′
**16S-L2021**	5′-CCTACCGAGCTTAGTAATAGCTGGTT-3′
**Reverse primers**	
**R0483**	5′-CCATGTTACGACTTGCCTCTTC-3′
**R1169**	5′-GTGGCTGCTTTTAGGCCCACT-3′
**R1624**	5′-CCCTCGTGATGCCGTTGATAC-3′
**Rend**	5′-GACCTGGATTACTCCGGTCTGA-3′
**16S-H-1**	5′-CTCCGGTCTGAACTCAGATCACGTAGG-3′
**16S-H2715**	5′-AAGCTCCATAGGGTCTTCTCGTC-3′

PCR products were visualized by agarose electrophoresis in the presence of ethidium bromide and consequently purified using 2 μL from a 1:4 dilution of ExoSapIt (Amersham, UK) per 5 μL of PCR product prior to cycle sequencing. Sequence data collection and visualization were performed on an ABI 3730xl automated sequencer (Applied Biosystems, USA) in Evrogen Inc., Moscow. The obtained sequences were aligned and deposited in GenBank under the accession numbers MG917762–MG917772 (Table 2).

**Table 2:** Sequences and voucher specimens of *Theloderma* and outgroup taxa used in this study

No.	Taxon	Specimen ID	GenBank accession No.	Country	Locality
**1**	*Rhacophorus schlegelii*	–	NC007178	Japan	Hiroshima
**2**	*Nasutixalus medogensis*	6255Rao	GQ285679	China	Xizang: Motuo
**3**	*Nyctixalus margaritifer*	TNHCJAM 3030	EU178087	Indonesia	Java
**4**	*Nyctixalus pictus*	FMNH 231095	DQ283133	Malaysia	Sabah: Lahad Datu
**5**	*Nyctixalus pictus*	FMNH 231094	GQ204777; GQ204726	Malaysia	–
**6**	*Nyctixalus pictus*	–	AF215349	Malaysia	–
**7**	*Nyctixalus pictus*	NMBE 1056413	JN705355; JN377342	Malaysia	Sarawak: Batang Ai
**8**	*Nyctixalus pictus*	MVZ 239460	GQ204783; GQ204732	Indonesia	–
**9**	*Nyctixalus pictus*	FMNH 231094-2	AF458135	Malaysia	–
**10**	*Nyctixalus pictus*	–	AF268255	Malaysia	–
**11**	*Nyctixalus pictus*	AH07001	GU154888	Malaysia	Sarawak: Gunung Mulu
**12**	*Nyctixalus spinosus*	pet trade	KT461916	Philippines	Mindanao
**13**	*Nyctixalus spinosus*	ACD 1043	DQ283114	Philippines	Mindanao
**14**	*Theloderma albopunctatum*	KIZ 060821217	EF564522	China	Guangxi: Jinxiu
**15**	*Theloderma albopunctatum*	KIZ 060821201	EF564521	China	Yunnan: Jinping
**16**	*Theloderma albopunctatum*	VNMN J2916	KJ802913	Vietnam	Vinh Phuc: Tam Dao
**17**	*Theloderma albopunctatum*	VNMN 3540	KJ802914	Vietnam	Lao Cai: Sa Pa
**18**	*Theloderma albopunctatum*	060821203Rao	GQ285677	China	Yunnan: Jinping
**19**	*Theloderma albopunctatum*	asperum-1	KT461884	Vietnam	Kon Tum: Kon Plong
**20**	*Theloderma albopunctatum*	asperum-2	KT461908	Vietnam	Kon Tum: Kon Plong
**21**	*Theloderma albopunctatum*	asperum-3	KT461909	Vietnam	Kon Tum: Kon Plong
**22**	*Theloderma albopunctatum*	ZMMU NAP-03557	KT461910	Vietnam	Hai Phong: Cat Ba
**23**	*Theloderma albopunctatum*	ZMMU NAP-03566	KT461911	Vietnam	Hai Phong: Cat Ba
**24**	*Theloderma albopunctatum*	ZMMU NAP-03575	KT461912	Vietnam	Hai Phong: Cat Ba
**25**	*Theloderma albopunctatum*	HN0806100	GQ285678	China	Hainan: Yinggeling
**26**	*Theloderma albopunctatum*	VNMN 4404	LC012854	Vietnam	Kon Tum: Ngoc Linh
**27**	*Theloderma albopunctatum*	VNMN 4405	LC012855	Vietnam	Gia Lai: Kon Ka Kinh
**28**	*Theloderma albopunctatum*	VNMN 4406	LC012856	Vietnam	Thanh Hoa: Xuan Lien
**29**	*Theloderma albopunctatum*	KUHE 23736	LC012858	Thailand	Doi Changdao
**30**	*Theloderma albopunctatum*	VNMN PAE262	LC012857	Vietnam	Son La: Ta Sua
**31**	*Theloderma albopunctatum*	VNMN J2888	LC012853	Vietnam	Vinh Phuc: Tam Dao
**32**	*Theloderma annae*	ZMMU NAP-05558	MG917766	Vietnam	Hoa Binh: Lac Son
**33**	*Theloderma asperum*	ZRC 1.1.9321	GQ204725; GQ204776	Malaysia	–
**34**	*Theloderma asperum*	pet trade	KT461929	Malaysia	Perak
**35**	*Theloderma auratum* **sp. nov.**	ZMMU A-5828	MG917767	Vietnam	Gia Lai: Kon Ka Kinh
**36**	*Theloderma auratum* **sp. nov.**	ZMMU A-5829	MG917768	Vietnam	Gia Lai: Kon Chu Rang
**37**	*Theloderma auratum* **sp. nov.**	ZMMU A-5830	MG917769	Vietnam	Gia Lai: Kon Chu Rang
**38**	*Theloderma auratum* **sp. nov.**	ZMMU A-5831	MG917770	Vietnam	Gia Lai: Kon Chu Rang
**39**	*Theloderma auratum* **sp. nov.**	ZMMU A-5832	MG917771	Vietnam	Kon Tum: Thac Nham
**40**	*Theloderma auratum* **sp. nov.**	ZMMU NAP-06402-2	MG917772	Vietnam	Kon Tum: Thac Nham
**41**	*Theloderma baibungense*	KIZ YPX37270	KU243080	China	Medog, Tibet
**42**	*Theloderma bicolor*	VNMN 1394	JX046475	Vietnam	Lao Cai: Sa Pa
**43**	*Theloderma bicolor*	bicolor-2	KT461923	Vietnam	Ninh Binh: Cuc Phuong
**44**	*Theloderma bicolor*	bicolor-3	KT461891; KT461899	Vietnam	Ninh Binh: Cuc Phuong
**45**	*Theloderma bicolor*	IEBR A.2011.4	JX046474	Vietnam	Lao Cai: Sa Pa
**46**	*Theloderma bicolor*	VNMN 3536	KJ802915	Vietnam	Lao Cai
**47**	*Theloderma corticale*	AMNH A161499	DQ283050	Vietnam	Vinh Phuc: Tam Dao
**48**	*Theloderma corticale*	IEBR 3267	JX046477	Vietnam	Vinh Phuc: Tam Dao
**49**	*Theloderma corticale*	corticale-1	KT461885	Vietnam	Ninh Binh: Cuc Phuong
**50**	*Theloderma corticale*	corticale-2	KT461886	Vietnam	Ninh Binh: Cuc Phuong
**51**	*Theloderma corticale*	IEBR E193.15	JX046476	Vietnam	Vinh Phuc: Tam Dao
**52**	*Theloderma corticale*	VNMN J2892	KJ802916	Vietnam	Tuyen Quang
**53**	*Theloderma corticale*	VNMN J2932	KJ802917	Vietnam	Vinh Phuc: Tam Dao
**54**	*Theloderma corticale*	JXDYS2015042501	KY290395	China	Guangxi: Jinxiu
**55**	*Theloderma corticale*	VNMN 3556	LC012841	Vietnam	Vinh Phuc: Tam Dao
**56**	*Theloderma corticale*	ZMMU NAP-06328	MG917764	Vietnam	Vinh Phuc: Tam Dao
**57**	*Theloderma corticale*	ZMMU NAP-05936	MG917765	Vietnam	Ha Tinh: Ke Go
**58**	*Theloderma gordoni*	VNMN 03013	JN688167	Vietnam	Nghe An
**59**	*Theloderma gordoni*	VNMN PAE217	KJ802918	Vietnam	Son La
**60**	*Theloderma gordoni*	KUHE 32447	KJ802919	Laos	Houaphan
**61**	*Theloderma gordoni*	VNMN 4407	LC012852	Vietnam	Kon Tum: Ngoc Linh
**62**	*Theloderma horridum*	LJT W44	KC465843	Malaysia	–
**63**	*Theloderma horridum*	LJT W45	KC465842	Malaysia	–
**64**	*Theloderma horridum*	ZMMU NAP-04015	KT461890	Thailand	Satun: Tha Le Ban
**65**	*Theloderma horridum*	KUHE 52582	LC012861	Malaysia	Negeri Sembilan, Kenaboi
**66**	*Theloderma lacustrinum*	NCSM84682	KX095245	Laos	Vientiane: Feuang, Nam Lik
**67**	*Theloderma lacustrinum*	NCSM84683	KX095246	Laos	Vientiane: Feuang, Nam Lik
**68**	*Theloderma laeve*	ZMMU NAP-01640	KT461928	Vietnam	Lam Dong: Cat Loc
**69**	*Theloderma laeve*	ZMMU NAP-01644	KT461907	Vietnam	Lam Dong: Cat Loc
**70**	*Theloderma laeve*	ZMMU NAP-01645	KT461913	Vietnam	Lam Dong: Cat Loc
**71**	*Theloderma laeve*	ZMMU NAP-02906	KT461883	Vietnam	Binh Phuoc: Bu Gia Map
**72**	*Theloderma laeve*	ZMMU NAP-02907	KT461905	Vietnam	Binh Phuoc: Bu Gia Map
**73**	*Theloderma laeve*	ZMMU NAP-02908	KT461906	Vietnam	Binh Phuoc: Bu Gia Map
**74**	*Theloderma laeve*	ZMMU NAP-03383	KT461892; KT461900	Vietnam	Lam Dong: Bao Loc
**75**	*Theloderma laeve*	ZMMU NAP-03408	KT461897; KT461898	Vietnam	Lam Dong: Bao Loc
**76**	*Theloderma laeve*	ZMMU NAP-03409	KT461920	Vietnam	Lam Dong: Bao Loc
**77**	*Theloderma lateriticum*	VNMN 1216	LC012851	Vietnam	Bac Giang: Yen Tu
**78**	*Theloderma lateriticum*	VNMN 1215	LC012850	Vietnam	Bac Giang: Yen Tu
**79**	*Theloderma lateriticum*	AMNH 168757	LC012848	Vietnam	Lao Cai: Sa Pa
**80**	*Theloderma lateriticum*	VNMN PAE 226	LC012849	Vietnam	Son La: Ta Sua
**81**	*Theloderma leporosum*	LJT W46	KC465841	Malaysia	–
**82**	*Theloderma leporosum*	leporosum-1	KT461922	Malaysia	Selangor
**83**	*Theloderma leporosum*	KUHE 52581	AB847128	Malaysia	Negeri Sembilan
**84**	*Theloderma licin*	KUHE 52599	KJ802920	Malaysia	Selangor
**85**	*Theloderma licin*	KUHE 19426	LC012859	Thailand	Nakon Sri Tamarat
**86**	*Theloderma moloch*	KIZ YPX31941	KU243081	China	Medog, Tibet
**87**	*Theloderma nebulosum*	ROM 39588	KT461887	Vietnam	Kon Tum: Ngoc Linh
**88**	*Theloderma nebulosum*	AMS R 173409	JN688168	Vietnam	Kon Tum: Ngoc Linh
**89**	*Theloderma nebulosum*	UNS00141	JN688169	Vietnam	Kon Tum: Ngoc Linh
**90**	*Theloderma nebulosum*	VNMN 39588	LC012845	Vietnam	Kon Tum: Ngoc Linh
**91**	*Theloderma palliatum*	ZMMU NAP-02757	KT461896; KT461904	Vietnam	Dak Lak: Chu Yang Sin
**92**	*Theloderma palliatum*	ZMMU NAP-02756	KT461930	Vietnam	Dak Lak: Chu Yang Sin
**93**	*Theloderma palliatum*	ZMMU NAP-02735	KT461926	Vietnam	Dak Lak: Chu Yang Sin
**94**	*Theloderma palliatum*	ZMMU NAP-02736	KT461927	Vietnam	Dak Lak: Chu Yang Sin
**95**	*Theloderma palliatum*	ZMMU NAP-02735	LC012843	Vietnam	Dak Lak: Chu Yang Sin
**96**	*Theloderma palliatum*	ZMMU NAP-02736	LC012844	Vietnam	Dak Lak: Chu Yang Sin
**97**	*Theloderma palliatum*	AMS R 173130	JN688172	Vietnam	Lam Dong: Bi Doup – Nui Ba
**98**	*Theloderma palliatum*	ZMMU NAP-01846	KT461893; KT461901	Vietnam	Lam Dong: Bi Doup – Nui Ba
**99**	*Theloderma palliatum*	ZMMU NAP-02511	KT461894; KT461902	Vietnam	Lam Dong: Bi Doup – Nui Ba
**100**	*Theloderma palliatum*	ZMMU NAP-02516	KT461895; KT461903	Vietnam	Lam Dong: Bi Doup-Nui Ba
**101**	*Theloderma petilum*	HNUE MNA.2012.0001	KJ802925	Vietnam	Dien Bien: Muong Nhe
**102**	*Theloderma phrynoderma*	CAS 247910	KJ128281	Myanmar	Tanintharyi
**103**	*Theloderma phrynoderma*	CAS 243920	KJ128280	Myanmar	Tanintharyi
**104**	*Theloderma pyaukkya* A	CAS 234857	KU244371	Myanmar	Chin
**105**	*Theloderma pyaukkya* A	CAS 234869	KU244370	Myanmar	Chin
**106**	*Theloderma pyaukkya* B	CAS 236133	KU244360	Myanmar	Kachin
**107**	*Theloderma pyaukkya* B	CAS 226113	KU244361	Myanmar	Kachin
**108**	*Theloderma rhododiscus*	AMNH A163892; A163893	DQ283392; DQ283393	Vietnam	Ha Giang: Tay Con Linh
**109**	*Theloderma rhododiscus*	KIZ060821063	EF564533	China	Guangxi: Jinxiu
**110**	*Theloderma rhododiscus*	KIZ060821170	EF564534	China	Guangxi: Jinxiu
**111**	*Theloderma rhododiscus*	SCUM 061102L	EU215530	China	Guangxi: Dayaoshan
**112**	*Theloderma rhododiscus*	CIB GX200807048	KJ802921	China	Guangxi
**113**	*Theloderma rhododiscus*	CIB GX200807017	LC012842	China	Guangxi
**114**	*Theloderma ryabovi*	ryabovi-1	KT461914	Vietnam	Kon Tum: Kon Plong: Mang Canh
**115**	*Theloderma ryabovi*	ryabovi-2	KT461915	Vietnam	Kon Tum: Kon Plong: Mang Canh
**116**	*Theloderma ryabovi*	VNMN 3924	LC012860	Vietnam	Kon Tum: Kon Plong: Mang Canh
**117**	*Theloderma stellatum*	stellatum-1	KT461918	Thailand	Chanthaburi: Phliu
**118**	*Theloderma stellatum*	ZMMU NAP-03961	KT461917	Thailand	Nakhon Nayok: Nang Rong
**119**	*Theloderma truongsonense*	ROM 39363	KT461925	Vietnam	Quang Binh: Phong Nha–Ke Bang
**120**	*Theloderma truongsonense*	ZMMU ABV-00301	KT461882	Vietnam	Khanh Hoa: Hon Ba
**121**	*Theloderma truongsonense*	ZMMU ABV-00319	KT461924	Vietnam	Khanh Hoa: Hon Ba
**122**	*Theloderma truongsonense*	AMS R 171510	JN688174	Vietnam	Quang Nam
**123**	*Theloderma truongsonense*	VNMN 4402	LC012847	Vietnam	Khanh Hoa: Hon Ba
**124**	*Theloderma truongsonense*	ZMMU NAP-07142	MG917762	Vietnam	Gia Lai: Kon Ka Kinh
**125**	*Theloderma truongsonense*	ZMMU NAP-07143	MG917763	Vietnam	Gia Lai: Kon Ka Kinh
**126**	*Theloderma vietnamense*	VNMN 3686	KJ802922	Vietnam	Phu Yen
**127**	*Theloderma vietnamense*	VNMN 3687	KJ802923	Vietnam	Phu Yen
**128**	*Theloderma vietnamense*	ZMMU NAP-00707	KT461889	Vietnam	Dong Nai: Nam Cat Tien
**129**	*Theloderma vietnamense*	ZMMU NAP-03680	KT461921	Vietnam	Tay Ninh: Lo Go – Xa Mat
**130**	*Theloderma vietnamense*	ZMMU NAP-03723	KT461919	Vietnam	Kien Giang: Phu Quoc
**131**	*Theloderma vietnamense*	ZMMU NAP-03724	KT461888	Vietnam	Kien Giang: Phu Quoc
**132**	*Theloderma vietnamense*	AMS R 173283	JN688170	Vietnam	Binh Thuan
**133**	*Theloderma vietnamense*	AMS R 174047	JN688171	Cambodia	Mondol Kiri
**134**	*Theloderma vietnamense*	KUHE 22056	LC012862	Thailand	MaeYom
**135**	*Theloderma* sp.	VNMN 4403	LC012846	Vietnam	Gia Lai: Mang Yang

–: Not available.

### Phylogenetic analyses

Sequences of the 12S rRNA, tRNA^val^, and 16S rRNA mtDNA fragments from 135 Rhacophoridae specimens, including 122 representatives of *Theloderma* (ca. 27 species), and 13 sequences of outgroup members of Rhacophoridae (genera *Rhacophorus*, *Nasutixalus*, and *Nyctixalus*) were included in the final alignment with a total length of 2 518 bp. Details on voucher specimens and GenBank accession Nos. used in phylogenetic analyses are summarized in Table 2. Nucleotide sequences were initially aligned using ClustalX 1.81 (Thompson et al., 1997) with default parameters, and then checked by eye in BioEdit 7.0.5.2 (Hall, 1999) and MEGA 7.0 (Kumar et al., 2016) and slightly adjusted.

The dataset was divided into three partitions, 12S rRNA, tRNA^val^, and 16S rRNA, with the optimal evolutionary models then estimated using MODELTEST v.3.06 (Posada & Crandall, 1998). For the 12S and 16S rRNA partitions, the best-fitting model according to the Akaike information criterion (AIC) was the HKY+I+G model; whereas for the tRNA^val^ partition, the Kimura 2-parameter model (+G+I) was selected as the one of best fit. Mean uncorrected genetic distances (*P*-distances) between sequences were determined with MEGA 7.0 (Kumar et al., 2016).

Matrilineal genealogy was inferred using Bayesian inference (BI) and maximum likelihood (ML) algorithms. The BI analyses were conducted in MrBayes 3.1.2 (Huelsenbeck & Ronquist, 2001; Ronquist & Huelsenbeck, 2003). Metropolis-coupled Markov chain Monte Carlo (MCMCMC) analyses were run with one cold chain and three heated chains for ten million generations and sampled every 1 000 generations. Five independent MCMCMC runs were performed and 1 000 trees were discarded as burn-in. Confidence in tree topology was tested by posterior probability (PP) for Bayesian inference (BI) trees (Huelsenbeck & Ronquist, 2001). Nodes with posterior probability values over 0.95 were *a priori* regarded as sufficiently resolved, whereas those between 0.95 and 0.90 were regarded as tendencies, and those below 0.90 were considered as unsupported.

The ML analyses were conducted using Treefinder (Jobb et al., 2004) and confidence in node topology was tested by non-parametric bootstrapping with 1 000 replicates (ML BS, see Felsenstein, 1985). We *a priori* regarded tree nodes with bootstrap (ML BS) values of 70% or greater and Bayesian posterior probabilities (BI PP) values over 0.95 as sufficiently resolved; ML BS values between 70% and 50% (BI PP between 0.95 and 0.90) were treated as tendencies and nodes with ML BS values below 50% (BI PP below 0.90) were regarded as unresolved (Felsenstein, 2004; Huelsenbeck & Hillis, 1993).

### Acoustic analyses

Advertisement calls of the *Theloderma* sp. were recorded in habitat in Kon Chu Rang Nature Reserve, Gia Lai Province, Tay Nguyen Plateau, Vietnam (N14°30${}^{\prime }$19.7${}^{\prime \prime }$, E108°32${}^{\prime }$30.0${}^{\prime \prime }$; 950 m a.s.l.) on 26 May 2016 from 0224 h to 0300 h at an ambient temperature of 21.5 °C (temperature was measured at the calling site immediately after recording with a digital thermometer KTJ TA218A Digital LCD Thermometer-Hydrometer) using a portable digital audio recorder Zoom h5 (ZOOM Corporation, Tokyo, Japan) in stereo mode with 48 kHz sampling frequency and 16-bit precision. In total, two recordings from two males (ZMMU A-5828 and A-5829) were made.

Calls were analyzed using Avisoft SASLab Pro software v.5.2.05 (Avisoft Bioacoustics, Germany). Before analysis, we reduced background noise using low-pass (up to 500 Hz) and high-pass filters (down to 7 kHz). All parameters were measured in the spectrogram window of Avisoft. Spectrograms were created under Hamming window, FFT-length 1 024 points, frame 75%, and overlap 87.5%. For figure spectrograms, we lowered the sampling rate to 22.05 kHz. Figure spectrograms were created under Hamming window, FFT-length 512 points, frame 100%, and overlap 50%. In total, we measured 214 *Theloderma* sp. calls.

We measured four temporal parameters (duration of each call and series and interval between successive calls and series) and three frequency parameters (initial and final fundamental frequency and frequency of maximum amplitude). We also calculated the call repetition rate (calls/s) by counting the number of calls within each series, minus one, and dividing that number by the series duration. All numeral parameters are given as means±*SE*, and the minimum and maximum values are given in parentheses (min–max).

## RESULTS

### Phylogenetic analyses

#### Sequences and statistics

The final alignment contained 2 518 aligned characters, with 1 090 conserved sites and 1 395 variable sites, of which 1 170 were found to be parsimony-informative. The transition-transversion bias (R) was 2.372 (data given for ingroup only). Nucleotide frequencies were 30.10% (A), 20.18% (T/U), 27.40% (C), and 22.31% (G).

### Position of *Theloderma* sp. in matrilineal genealogy

**Figure 2:**
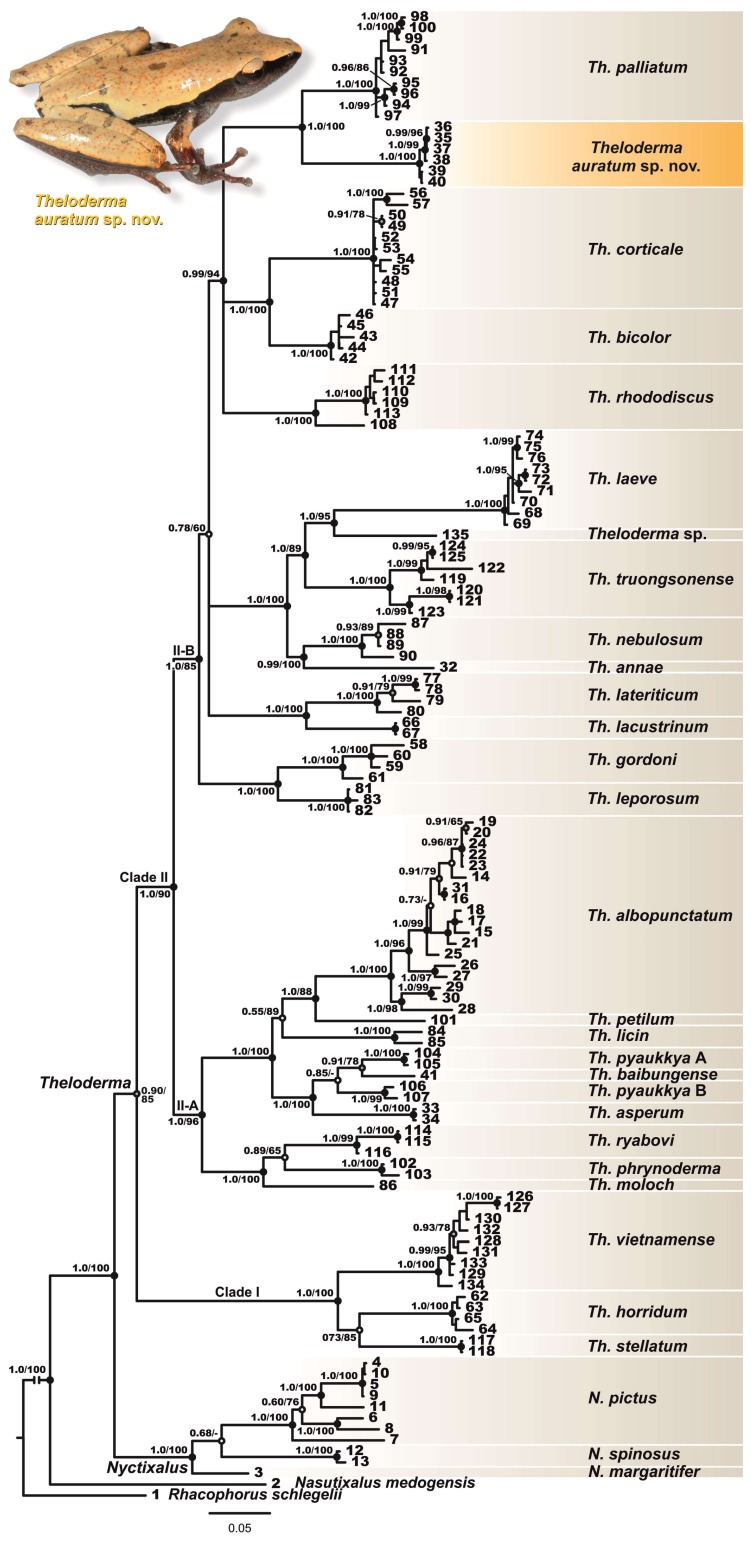
Bayesian inference dendrogram of *Theloderma* and its relatives derived from analysis of 2 518-bp length 12SrRNA – 16S rRNA mtDNA gene fragments

The phylogenetic analyses results are presented in Figure 2. The ML and BI phylogenetic analyses resulted in essentially similar topologies. In general, the BI cladogram topology was consistent with results reported in previous work (Nguyen et al., 2015; Sivongxay et al., 2016), suggesting monophyly of the clade joining *Nyctixalus* and *Theloderma* (node support values 1.0/100, hereafter given for BI PP/ML BS, respectively) and monophyly of *Theloderma*, though only with moderate support (0.90/85).

Numbers of specimens (No. 1–135), corresponding voucher specimen information and GenBank accession numbers are given in Table 2. BI PP/ML BS support values are given only for resolved nodes. Highly supported nodes are marked with black circles (PP node support >0.95), grey circles indicate moderately supported nodes (0.90<PP<0.95), and white circles indicate unsupported nodes (PP<0.90). Photo by Nikolay A. Poyarkov.

The genus *Theloderma* was divided into two strongly-supported major clades: Clade I (1.0/100), joining *Th. stellatum*, *Th. Horridum*, and *Th. vietnamense* Poyarkov, Orlov, Moiseeva, Pawangkhanant, Ruangsuwan, Vassilieva, Galoyan, Nguyen & Gogoleva, 2015 (corresponding to the subgenus *Stelladerma*) and Clade II (1.0/90), joining all remaining species of the genus (subgenus *Theloderma* s.str.) (Figure 2). Clade II was further subdivided into subclade II-A (Sundaland, Indochina, eastern Himalayas, southern China; 1.0/96) and subclade II-B (Indochinese species; 1.0/85).

Subclade II-A joined two species groups: the *Th. moloch* group consisting of large-sized warty species from Indochina and the eastern Himalayas (i.e., *Th. moloch*, *Th. phrynoderma* (Ahl, 1927), and *Th. ryabovi* Orlov, Dutta, Ghate & Kent, 2006; 1.0/100)) and the *Th. asperum* group consisting of medium to small-sized species with contrasting black and white dorsal coloration (i.e., *Th. asperum* (Boulenger, 1886), *Th. pyaukkya* Dever, 2017, *Th. baibungense* (Jiang, Fei & Huang, 2009), *Th. licin* McLeod & Ahmad, 2007, *Th. petilum* (Stuart & Heatwole, 2004), and *Th. albopunctatum* (Liu & Hu, 1962); 1.0/100)). Our data strongly suggested paraphyly of *Th. pyaukkya* with respect to *Th. baibungense*, with the former subdivided into two divergent lineages from northern and central Myanmar (A and B, see Figure 2) with poorly resolved genealogical relationships. Furthermore, *Th. albopunctatum* represents a species complex with at least four divergent lineages with unclear geographic structuring (see Figure 2, Table 2).

Phylogenetic relationships within subclade II-B were essentially unresolved (Figure 2), though the following species groups were supported: *Th. leporosum* group (joining *Th. leporosum* Tschudi, 1838 and *Th. gordoni* Taylor, 1962; 1.0/100), *Th. lateriticum* group (including *Th. lateriticum* Bain, Nguyen & Doan, 2009 and *Th. lacustrinum* Sivongxay, Davankham, Phimmachak, Phoumixay & Stuart, 2016; 1.0/100), *Th. laeve* group (including *Th. laeve*, *Th. truongsonense* (Orlov & Ho, 2005), *Th. nebulosum* Rowley, Le, Hoang, Dau & Cao, 2011, and *Th. annae* Nguyen, Pham, Nguyen, Ngo & Ziegler, 2016; 1.0/100), and *Th. corticale* group (joining *Th. corticale* (Boulenger, 1903), *Th. bicolor* (Bourret, 1937), *Th. rhododiscus* (Liu & Hu, 1962), *Th. palliatum*, and *Theloderma* sp. from the Tay Nguyen Plateau; 0.99/94). A single specimen (VNMN 4403) from Gia Lai Province identified as *Th. laeve* by Nguyen et al. (2015) was grouped with samples of *Th. laeve* s. str. from southern Vietnam with high support (1.0/95) but represented a clearly distinct genealogical lineage and was identified as *Theloderma* sp. in the present paper.

The *Theloderma* sp. from Tay Nguyen Plateau was recovered as a sister species of *Th. palliatum*, which inhabits the montane forests of Langbian Plateau, with strong node support (1.0/100) (Figure 2); however, no clear structuring was observed within the *Theloderma* sp. clade.

### Sequence divergence

The uncorrected *P*-distances among and within the studied 2 518-bp mtDNA fragments for the examined *Theloderma* species are shown in Table 3 (data given for the ingroup only). The interspecific uncorrected genetic *P*-distances between the *Theloderma* sp. from Tay Nguyen Plateau and other congeners varied from 8.9% (between *Theloderma* sp. and sister species *Th. palliatum*) to 17.9% (between *Theloderma* sp. and *Th. laeve*) (Table 3). This degree of pairwise divergence was high, notably greater than the genetic divergence thresholds representing species level differentiation in frogs (Vences et al., 2005a, 2005b; Vieites et al., 2009).

**Table 3:** Uncorrected *P*-distance (percentage) between 12S rRNA – 16S rRNA 2 524 bp fragment sequences of *Theloderma* species included in phylogenetic analyses (below the diagonal) and standard error estimates (above the diagonal)

	Species	1	2	3	4	5	6	7	8	9	10	11	12	13	14	15	16	17	18	19	20	21	22	23	24	25	26
**1**	*Th. albopunctatum*	***2.5***	2.0	1.7	1.9	1.9	1.9	2.1	2.0	2.1	1.9	1.9	1.8	2.0	1.6	1.5	2.0	2.1	1.7	2.0	1.7	1.9	1.8	2.2	1.7	2.0	1.8
**2**	*Th. annae*	17.1	**–**	2.1	1.8	1.9	1.7	1.7	1.7	1.9	1.8	2.1	1.7	1.6	1.9	1.9	1.4	1.9	2.1	2.0	1.7	1.7	1.8	1.8	1.7	1.8	1.8
**3**	*Th. asperum*	12.1	17.6	***0.0***	2.1	1.6	1.8	1.8	1.8	2.2	1.9	1.9	1.8	1.9	1.8	1.7	1.7	2.1	1.8	1.9	1.5	2.0	1.8	2.3	1.7	2.0	1.6
**4**	*Th. auratum* **sp. nov.**	**13.9**	**14.8**	**15.1**	***0.4***	2.0	1.8	1.7	1.5	2.1	1.6	1.9	1.8	1.8	2.1	1.9	1.9	1.7	1.9	2.0	1.9	1.5	1.7	2.0	1.6	1.9	1.9
**5**	*Th. baibungense*	11.5	15.5	8.1	**15.1**	**–**	1.7	1.7	1.6	1.8	1.7	2.0	1.7	1.9	1.7	1.8	1.7	1.8	1.8	1.8	1.1	1.9	1.8	2.0	1.7	2.0	1.8
**6**	*Th. bicolor*	12.7	12.2	13.0	**9.2**	13.0	***0.3***	1.3	1.5	1.9	1.5	2.1	1.4	1.5	1.9	1.5	1.7	1.7	1.8	1.9	1.6	1.4	1.8	2.1	1.5	1.9	1.8
**7**	*Th. corticale*	15.2	12.6	13.8	**9.9**	13.5	6.1	***0.7***	1.4	2.1	1.8	1.9	1.5	1.4	2.0	1.6	1.5	1.6	2.1	1.8	1.7	1.5	1.6	2.1	1.6	1.6	1.6
**8**	*Th. gordoni*	14.0	14.3	14.1	**11.9**	11.1	9.5	10.6	***3.3***	1.9	1.5	2.0	1.5	1.3	1.9	1.5	1.5	1.8	1.8	1.8	1.5	1.5	1.5	2.0	1.7	2.0	1.7
**9**	*Th. horridum*	18.3	18.4	18.3	**16.3**	18.0	14.0	15.0	15.7	***0.6***	1.8	2.3	1.8	1.8	1.9	1.9	2.2	2.2	1.9	2.0	1.7	1.8	1.9	1.7	1.8	1.6	2.1
**10**	*Th. lacustrinum*	14.4	12.2	14.9	**10.8**	14.2	8.4	11.3	11.9	14.7	***0.0***	2.1	1.2	1.7	1.8	1.6	1.7	1.8	2.0	2.1	1.6	1.4	1.8	1.8	1.7	1.9	1.9
**11**	*Th. laeve*	13.8	17.1	15.6	**17.9**	15.5	16.6	16.4	15.8	21.0	16.6	***1.1***	2.2	2.2	1.8	2.0	1.8	2.1	2.2	1.7	1.8	2.0	1.5	2.3	1.6	2.2	1.6
**12**	*Th. lateriticum*	15.6	12.8	17.2	**12.5**	14.7	9.1	11.0	13.7	15.6	6.7	16.5	***3.6***	1.4	1.8	1.5	1.7	1.6	1.9	2.1	1.6	1.6	1.9	1.8	1.7	1.9	1.8
**13**	*Th. leporosum*	13.8	13.9	14.2	**11.1**	11.5	7.9	8.6	8.3	15.3	10.8	15.8	11.1	***0.0***	1.9	1.4	1.7	1.9	2.0	1.8	1.7	1.7	1.8	2.2	1.6	1.9	1.6
**14**	*Th. licin*	11.2	16.6	11.2	**14.7**	11.7	12.1	14.2	15.1	17.0	13.7	16.4	15.1	14.2	***3.4***	1.8	1.9	2.1	1.7	2.1	1.6	1.8	1.7	2.1	1.7	1.9	1.9
**15**	*Th. moloch*	7.7	9.5	6.8	**9.0**	7.7	6.5	8.3	7.7	12.4	7.7	11.3	8.8	6.4	8.4	**–**	1.6	1.7	1.9	1.6	1.4	1.5	1.4	2.3	1.5	2.1	1.7
**16**	*Th. nebulosum*	16.3	11.1	14.0	**11.3**	13.5	10.1	10.6	12.8	18.2	13.2	12.9	12.1	11.1	14.3	9.1	***1.7***	2.0	2.1	1.7	1.4	1.7	1.8	2.3	1.4	2.1	1.5
**17**	*Th. palliatum*	14.9	14.9	15.4	**8.9**	13.6	7.7	9.2	12.2	16.2	11.1	18.5	10.6	11.3	15.5	9.5	13.6	***0.9***	2.0	2.3	1.7	1.7	1.9	2.2	1.9	2.2	1.9
**18**	*Th. petilum*	9.6	16.6	12.2	**14.2**	9.8	12.3	15.3	13.3	16.4	14.6	14.3	14.5	12.5	12.0	7.3	15.3	15.3	**–**	2.1	1.6	1.9	2.1	2.0	1.8	2.0	2.2
**19**	*Th. phrynoderma*	11.6	12.8	11.3	**12.8**	11.7	12.2	11.4	11.6	17.7	15.1	12.5	15.9	9.8	13.2	5.9	11.1	14.5	12.5	***1.1***	1.5	2.0	1.4	2.4	1.6	2.0	1.7
**20**	*Th. pyaukkya*	11.1	14.3	8.5	**14.0**	5.4	10.4	13.2	11.3	17.4	12.6	14.7	13.9	11.1	10.0	6.4	12.2	12.3	9.9	10.2	***3.9***	1.7	1.7	2.0	1.5	1.9	1.6
**21**	*Th. rhododiscus*	13.7	15.3	13.0	**9.8**	12.6	8.4	10.7	10.0	14.6	10.0	15.3	11.8	10.1	13.4	6.2	13.2	10.6	12.7	14.8	11.0	***1.2***	1.6	2.1	1.5	2.0	1.8
**22**	*Th. ryabovi*	14.4	16.6	14.2	**14.1**	13.4	13.9	13.5	13.0	19.8	14.1	12.6	15.7	13.2	14.1	6.4	14.1	15.1	14.5	9.3	11.6	12.4	***2.5***	2.2	1.7	2.1	1.6
**23**	*Th. stellatum*	18.4	17.2	18.6	**16.5**	18.9	14.7	15.0	18.1	9.5	15.5	21.7	16.2	16.9	17.8	13.2	18.4	16.2	16.9	20.5	18.2	15.4	22.1	***0.0***	1.9	1.6	2.2
**24**	*Th. truongsonense*	15.1	12.6	13.8	**13.4**	15.1	11.7	13.0	14.6	17.7	12.0	12.8	13.7	13.4	15.3	8.6	10.5	15.0	14.7	11.9	13.8	13.4	14.9	16.2	***3.8***	1.9	1.5
**25**	*Th. vietnamense*	17.4	15.4	18.2	**15.2**	18.2	13.1	12.6	15.7	10.6	14.5	19.4	15.2	15.0	16.7	12.4	16.7	15.8	17.4	19.3	17.1	14.5	19.4	9.7	15.8	***1.6***	2.0
**26**	*Theloderma* sp.	12.7	12.4	12.1	**13.4**	12.8	11.4	11.9	12.1	18.0	12.4	9.0	12.4	10.7	13.8	6.9	8.3	14.1	14.2	9.7	11.0	12.1	10.6	18.3	8.9	16.3	**–**

–: Not available.

### Taxonomy

Based on the phylogenetic analyses of the 2 518-bp length 12S rRNA and 16S rRNA mtDNA fragment sequences, the examined specimens of *Theloderma* sp. from Tay Nguyen Plateau in central Vietnam represented a highly divergent mtDNA lineage, clearly distinct from all other *Theloderma* species for which comparable mtDNA sequences are available (Figure 2), and a sister species of *Th. palliatum* from Langbian Plateau of southern Vietnam. The observed differences in mtDNA sequences were congruent with evidence from the diagnostic morphological characters (see “Comparisons”). These results support our hypothesis that the small-sized “smooth” *Theloderma* sp. from Tay Nguyen Plateau represents a previously unknown species, which we describe herein.

*Theloderma auratum* sp. nov.

**Figure 3:**
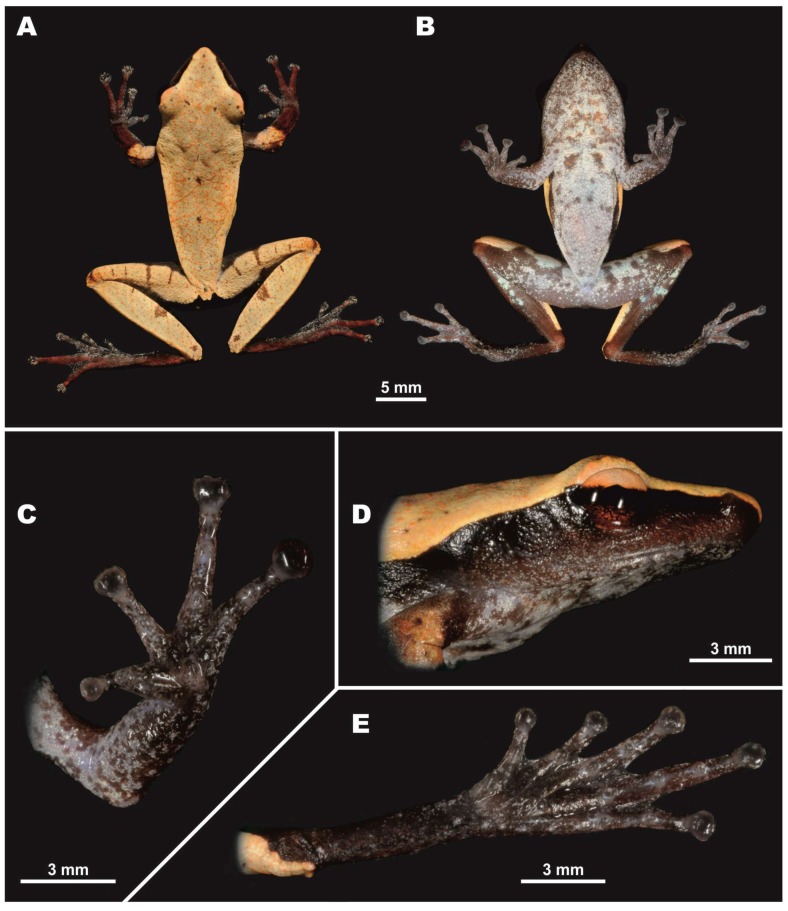
Male holotype of *Theloderma auratum* sp. nov. (ZMMU A-5828) in life (*ex situ*)

**Figure 4:**
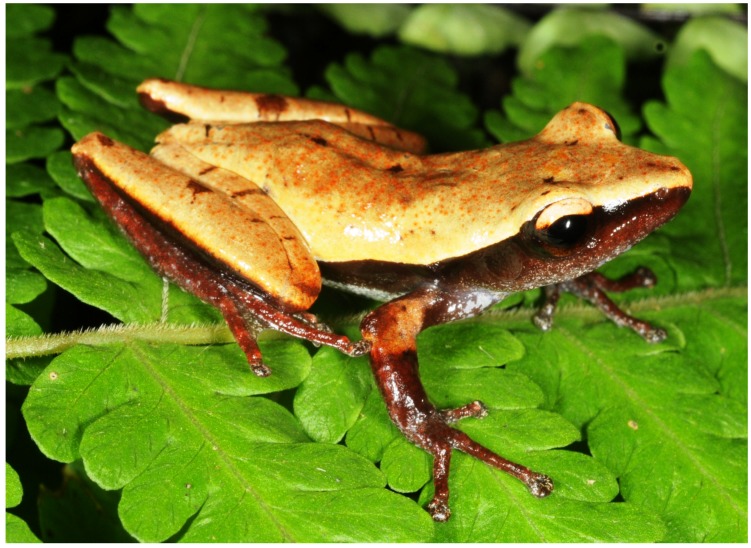
Dorsolateral view of male holotype of *Theloderma auratum* sp. nov. (ZMMUA-5828) in life (*in situ*) (Photo by Nikolay A. Poyarkov)

**Figure 5:**
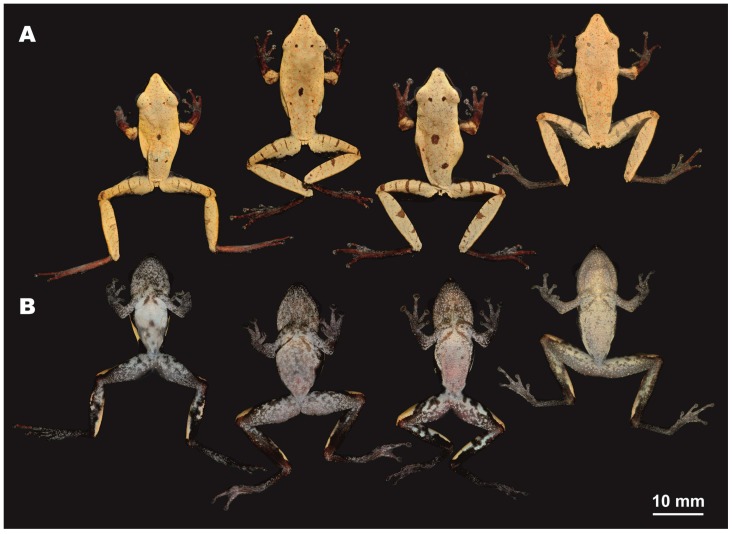
Male paratypes of *Theloderma auratum* sp. nov. in life in dorsal (A) and ventral (B) views (*ex situ*)

**Figure 6:**
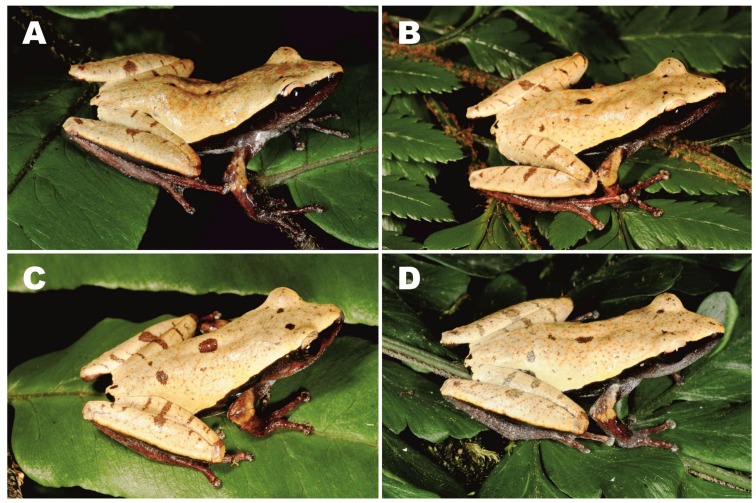
Dorsolateral views of male paratypes of *Theloderma auratum* sp. nov. in life (*in situ*)

**Figure 7:**
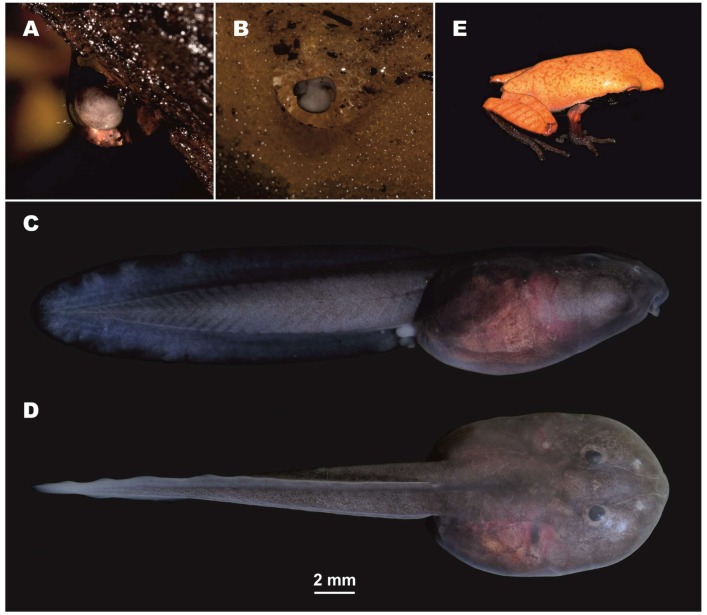
Ontogenetic stages of *Theloderma auratum* sp. nov. in life (*ex situ*)

**Figure 8:**
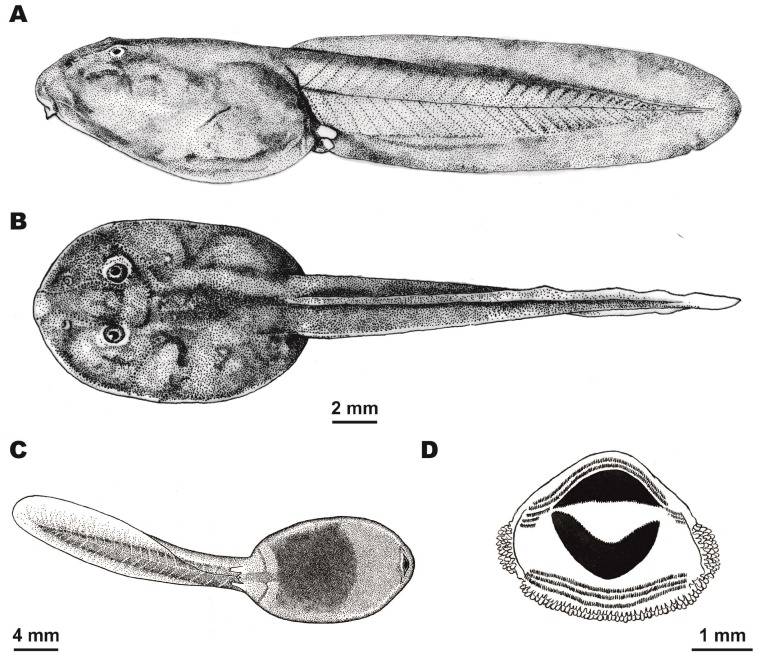
Tadpole morphology of *Theloderma auratum* sp. nov. (Gosner stage 31)

Table 4, Table 5; Figure 3, Figure 4, Figure 5, Figure 6, Figure 7 and Figure 8.

**Table 4:** Measurements of the type series of *Theloderma auratum* sp. nov. (all in mm)

**Specimen ID**	**Sex**	**SVL**	**A-G**	**HW**	**HL**	**HD**	UEW	**IOD**	**ED**	**TD**	**ESL**	**IND**	**END**	**TED**	**NS**	**FLL**
ZMMU A-5828 holotype	M	25.1	11.9	7.9	9.5	4.0	2.2	3.0	3.4	1.8	4.3	2.3	3.0	0.8	1.8	12.7
ZMMU A-5829 paratype	M	25.8	11.9	8.1	10.4	4.2	2.3	2.9	3.3	2.0	4.7	2.4	2.8	0.8	2.0	14.1
ZMMU A-5830 paratype	M	25.7	11.8	8.0	10.2	4.1	2.5	3.1	3.4	1.9	4.7	2.3	2.8	0.7	1.7	13.7
ZMMU A-5831 paratype	M	21.8	11.2	7.7	9.2	3.6	2.1	3.0	3.2	1.7	4.3	2.3	2.8	0.6	1.9	12.4
ZMMU A-5832 paratype	M	26.1	12.3	8.2	10.2	4.3	2.2	3.1	3.4	2.0	4.6	2.4	2.9	0.7	1.7	14.5
		**LAL**	**ML**	**FFL**	**TFL**	**FTD**	**NPL**	**MCTe**	**HLL**	**FL**	**TL**	**FOT**	**FTL**	**FFTL**	**HTD**	**MTTi**
ZMMU A-5828 holotype	M	5.6	6.8	2.4	5.0	1.3	1.1	0.8	40.0	11.9	13.7	11.0	1.3	5.4	1.2	1.1
ZMMU A-5829 paratype	M	5.7	7.5	2.3	4.8	1.4	1.9	0.8	41.5	12.3	14.1	10.9	1.4	5.4	1.3	1.3
ZMMU A-5830 paratype	M	4.8	6.7	2.2	4.8	1.3	1.4	0.6	40.0	12.4	14.3	10.7	1.4	5.4	1.2	1.0
ZMMU A-5831 paratype	M	5.0	6.2	2.3	4.4	1.3	1.1	0.7	36.1	11.0	12.5	10.4	1.6	5.3	1.0	1.1
ZMMU A-5832 paratype	M	5.8	6.9	2.3	4.9	1.2	1.5	0.8	39.1	11.9	14.1	10.7	1.3	5.4	0.9	1.1

For abbreviations, see “Materials and methods”.

**Table 5:** Main morphometric parameters of *Theloderma auratum* sp. nov. tadpoles (*n*=5; all in mm); developmental stages based on tables of Gosner (1960)

Characters	ZISP 13422 (Stage 29)	ZISP 13423 (Stage 30)	ZISP 13424 (Stage 33)	ZISP 13425 (Stage 35)	ZISP 13426 (Stage 36)
BH	6.2	6.2	6.3	6.6	6.9
BL	11.4	12.8	11.9	13.3	13.0
BW	8.3	8.4	8.1	9.3	9.6
ED	1.0	1.3	1.2	1.3	1.7
IOD	3.4	3.4	3.0	3.2	3.9
LF	1.8	2.1	2.0	2.1	2.0
MTH	4.7	4.6	5.1	5.9	5.7
NN	2.0	2.1	2.3	2.0	2.2
NP	2.0	2.4	2.6	2.3	2.3
ODW	3.2	3.7	3.2	3.7	3.6
RN	2.1	1.7	2.1	2.1	2.2
SS	8.5	8.6	8.2	9.3	9.2
TAL	18.5	19.6	20.3	22.7	23.6
TL	29.9	32.4	32.2	36.0	36.6
TMH	3.5	3.1	3.4	3.4	3.9
TMW	3.0	2.8	3.0	3.4	3.5
UF	1.6	1.8	2.0	2.5	2.0

For abbreviations, see “Materials and methods”.

A: Dorsal view; B: Ventral view; C: Volar view of left hand; D: Lateral view of head; E: Plantar view of left foot. Photos by Nikolay A. Poyarkov.

From left to right: A-5831, A-5829, A-5830, and A-5832. Scale bar, 10 mm. Photos by Nikolay A. Poyarkov.

A: A-5831; B: A-5829; C: A-5830; D: A-5832. Photos by Nikolay A. Poyarkov.

A: Freshly laid egg; B: Developing embryo; C: Tadpole (Gosner stage 31), lateral view; D: Tadpole (Gosner stage 31), dorsal view; E: Metamorph. Photos A–B and E by Ivan I. Kropachev; photos C–D by Nikolay A. Poyarkov.

A: Lateral view; B: Dorsal view; C: Ventral view; D: Oral disk. Figures A–B by Valentina D. Kretova, Figures C–D by Ivan I. Kropachev.

**Holotype**: ZMMU A-5828 (field number NAP-06351), adult male from montane evergreen tropical forest in Kon Chu Rang Nature Reserve, Gia Lai Province, Tay Nguyen Plateau, central Vietnam (N14°30${}^{\prime }$19.7${}^{\prime \prime }$, E108°32${}^{\prime }$30.0${}^{\prime \prime }$; elevation 950 m a.s.l.), collected when calling from bush leaves ca. 40 cm above the ground on 26 May 2016 at 0235 h by Nikolay A. Poyarkov.

**Paratypes**: ZMMU A-5829 (field number NAP-06352) and ZMMU A-5830 (field number NAP-06353), two adult males from the same locality and with the same collection information as the holotype; ZMMU A-5831 (field number NAP-06354), adult male from montane evergreen tropical forest in Kon Ka Kinh National Park, Gia Lai Province, Tay Nguyen Plateau, central Vietnam (N14°13${}^{\prime }$02.8${}^{\prime \prime }$, E108°19${}^{\prime }$53.3${}^{\prime \prime }$; elevation 1 420 m a.s.l.), collected ca. 1 m above the ground from vegetation on 12 May 2016 at 2300 h by Nikolay A. Poyarkov; ZMMU A-5832 (field number NAP-06402), adult male from montane evergreen tropical forest in Thac Nham Forest, Kon Plong District, Mang Canh Commune, Kon Tum Province, Tay Nguyen Plateau, Vietnam (N14°43${}^{\prime }$32.4${}^{\prime \prime }$, E108°18${}^{\prime }$04.5${}^{\prime \prime }$; elevation 1 230 m a.s.l.), collected ca. 30 cm above the ground from vegetation on 5 June 2016 at 2000 h by Nikolay A. Poyarkov. 

**Referred specimens**: ZISP 13422–13426, five tadpoles (Gosner stages 29–36) born and reared in captive laboratory conditions from adult specimens collected from montane evergreen tropical forest in Kon Plinh Village, Kon Plong District, Kon Tum Province, Tay Nguyen Plateau, Vietnam; elevation ca. 1 000 m a.s.l.) in June 2014 by Ivan I. Kropachev and Nikolai L. Orlov.

**Diagnosis**: The new species was assigned to the genus *Theloderma* by its (1) distinct tympanum, (2) terminal phalanx with Y-shaped distal end, (3) intercalary cartilage between terminal and penultimate phalanges of digits, (4) tips of digits expanded into large disks bearing circummarginal grooves, (5) head skin not co-ossified to skull (Liem, 1970; Nguyen et al., 2015; Poyarkov et al., 2015; Rowley et al., 2011), and molecular data (Figure 2). *Theloderma auratum* **sp. nov.** is distinguished from all other *Theloderma* by a combination of the following morphological attributes: (1) absence of bony ridges from canthus rostralis to occiput; (2) completely smooth skin on dorsum lacking calcified warts or asperities; (3) pointed elongated tapering snout with distinct rounded canthus rostralis, nostrils dorsolateral, not protuberant; (4) vocal opening in males absent; (5) vomerine teeth absent; (6) small body size in males (SVL 21.8–26.4 mm); (7) head longer than wide, head width to head length ratio 78%–84%; eye diameter to SVL ratio 13%–15%; snout length to SVL ratio 16%–20%; (8) comparatively small tympanum: tympanum diameter half of eye diameter (TD/EL ratio 50%–60%), tympanum with few tiny tubercles; (9) supratympanic fold absent; (10) ventral surfaces completely smooth; (11) webbing between fingers absent; (12) external and internal metacarpal tubercles present, supernumerary metacarpal tubercle single, medial, and oval in shape; (13) toes half-webbed, toe-webbing formula: I 2–2¼ II 1½–2¾ III 2–3¼ IV 3–1½ V; (14) inner metatarsal tubercle present, oval; outer metatarsal tubercle absent; (15) iris bicolored, golden-orange dorsally, black with copper-red flecks ventrally with a wide black longitudinal stripe running medially, incorporating black pupil; (16) dorsal surfaces golden-yellow with sparse golden-orange speckling or reticulations and few small dark-brown spots (usually located at mid-dorsum, on upper eyelids, snout tip, and sacral area); (17) lateral sides of head and body with wide dark reddish-brown to black lateral stripes, clearly separated from lighter dorsal coloration by straight contrasting edge; (18) ventral surfaces of body, throat, and chest greyish-blue with indistinct brown confluent blotches; (19) upper eyelids with few (3–5) very small flat reddish superciliary tubercles; (20) limbs dorsally reddish-brown, ventrally brown with small bluish-white speckles.

The new species is also markedly distinct from all congeners for which comparable sequences of the 2 518-bp length 12S rRNA to 16S rRNA mitochondrial DNA fragments are available (uncorrected genetic distance *P*>8.9%). 

**Description of holotype**: Small-sized rhacophorid frog specimen in a good state of preservation; body slender, dorsoventrally compressed (Figure 3). Skin on ventral surface of left femur of holotype medially dissected 5 mm, with femoral muscles (partial) removed for molecular genetic analyses. Measurements of holotype are given in Table 4 (in mm).

**Head**: Head much longer than wide (HW/HL 83%), quite deep (HD/HL 42%), moderately flattened; dorsally smooth with skin not co-ossified to skull, lacking calcified warts, asperities, or sculpturing; snout long (ESL/HL 45%) and tapering, snout tip pointed in dorsal view (Figure 3A), rounded in profile (Figure 3D), snout notably projecting beyond margin of lower jaw (Figure 3D); nostril ovoid, not protuberant (Figure 3D), oriented dorsolaterally, located much closer to tip of snout than to eye (END/ESL 69%) (Figure 3D); canthus rostralis distinct, rounded; loreal region slightly concave; eyes large (ED/HL 36%), eye diameter less than snout length (ED/ESL 78%), notably protuberant in dorsal view (Figure 3A) and profile (Figure 3D), pupil horizontal, ovoid (Figure 3D; Figure 4); tympanum distinct and rounded, with vertical tympanum diameter equal to horizontal (TD) diameter (ratio 1.0); tympanic rim distinct, slightly elevated above skin of temporal region (Figure 3D), tympanum comparatively small comprising half of eye diameter (TD/ED 53%), located close to eye (TED/ED 22%); pineal ocellus absent; vomerine teeth absent; choanae ovoid, almost hidden under margins of mouth roof; vocal sac openings not discernable; tongue wide, spatulate, attached anteriorly with free posterior end, with no distinct notch at posterior end; supratympanic fold indistinct, smooth (Figure 3D, Figure 4).

**Forelimbs**: Forelimbs thin, slender; relative finger lengths: I<II<IV<III; tips of all fingers with well-developed disks with distinct circummarginal grooves (Figure 3C), disks two times wider than finger widths (third finger disk width (FTD) 205% of third finger width at penultimate phalanx), disks rounded to slightly trapezoid, slightly expanded transversally, third finger disk width 125% of third finger disk length; third finger disk width (FTD) 72% of tympanum diameter (TD); dermal fringing on fingers not developed (Figure 3C); finger webbing absent; subarticular tubercles rather large, rounded, slightly protruding, distinct on all fingers, finger subarticular formula: I (1), II (1), III (2), IV (2); supernumerary metacarpal (palmar) tubercle single, medial, and ovoid; nuptial pad present, ovoid, elongated, covering prepollex area, comprising 46% of finger I length; two metacarpal tubercles present, inner metacarpal small and rounded, outer metacarpal tubercle flattened (Figure 3C). 

**Hindlimbs**: Hindlimbs slender, relatively long, heels overlap when legs are at right angles to body (Figure 3A), tibiotarsal articulations reach well beyond tip of snout; tibia slightly over half of snout-vent length (TL/SVL ratio 55%); dermal ridge along outer side of tibia or tarsal fold absent; toes half-webbed, toe-webbing formula: I 2–2¼ II 1½–2¾ III 2–3¼ IV 3–1½ V; weak dermal fringes reaching to disks of all toes. Tips of toes bearing small disks with distinct circummarginal and transverse grooves; disks rounded, notably smaller than those of fingers (HTD/FTD ratio 91%); fourth toe disk width (HTD) 105% of fourth toe disk length; relative toe lengths: I<II<V<III<IV; round, clearly distinct protuberant subarticular tubercles on all toes, toe subarticular formula: I (1), II (1), III (2), IV (3), V (2); inner metatarsal tubercle well pronounced and notably protuberant, oval-shaped, 2.1 times longer than wide, outer metatarsal tubercle or supernumerary tubercles absent (Figure 3E). 

**Skin texture and skin glands**: Dorsal skin smooth, with numerous small flat tubercles irregularly scattered on dorsal surfaces of head and body, forming weak reticulate pattern in sacral area (Figure 3A), lateral surfaces of head and body completely smooth; tympanum with few tiny evenly scattered tubercles; upper eyelids with few (3–5) very small flat reddish superciliary tubercles (Figure 3D); calcified asperities and warts on dorsum absent; area above insertion of forelimbs, and lateral sides of belly smooth; supratympanic and dorsolateral folds absent; dorsal surface of limbs weakly shagreened, ventral sides of limbs smooth; throat and chest smooth, belly and ventral surface of thigh smooth; dermal appendage at vent, dermal fringes and dermal tibiotarsal projections absent. 

**Color of holotype in life**: Background of dorsal surface of head, body, forearms, thighs, and shanks uniform golden-yellow with numerous tiny flat golden-orange tubercles on dorsal surfaces and supraciliary tubercles, getting somewhat denser posteriorly and forming weak reticulations in sacral area (Figure 3A; Figure 4). Small dark-brown spots of irregular shape located on dorsal surface of snout between nostrils, upper eyelids (two small spots on left and four spots on right upper eyelid), two spots on orbit margins between eyes, few small brown flecks in scapular area, two larger brown spots on mid-dorsal line (one in middle of dorsum, one in sacral area) and a single small spot above cloaca (Figure 3A). Elbows dorsally dark orange-brown. Four dark transverse dark-brown lines on dorsal surface of each thigh, orange-brown spot on each knee; on shanks a single short brown line at proximal end, larger light-brown blotch medially and smaller brown spot at distal end near tibiotarsal joint (Figure 3A).

Lateral sides of head and body with wide dark reddish-brown to black lateral stripes, clearly separated from lighter dorsal coloration by straight contrast edge, running from snout tip along canthus rostralis to anterior eye corner, then from posterior eye corner above tympanum and posteriorly to groin; dorsal edge of wide dark lateral stripe sharp and straight until groin, which shows very shallow dark inguinal loop with rounded edges. Tympanum uniformly dark-brown with no markings; tympanal area ventrally reddish-brown, dorsally black, sharply edged from light golden-yellow supratympanic area (Figure 3D).

Background of dorsal surfaces of arms, hands, and feet reddish-brown, with dense bluish-white to turquoise speckles, which spread to fingers and toes (Figure 3A); disks on digits dorsally brownish with dense whitish marbling, ventrally grey. Ventral surfaces of body, including belly and chest greyish-blue with indistinct brown confluent blotches, getting smaller and denser anteriorly, forming dense brownish pattern on throat (Figure 3B). Two oblique brownish spots in chest area. Ventral surfaces of forelimbs grey with whitish speckling (Figure 3B, C). Ventral surfaces of hindlimbs reddish-brown with large bluish to turquoise blotches on groin, femur, and medial part of shank (Figure 3B). Throat, chest, and ventral surfaces of thighs with small bluish-white speckles.

Pupil horizontal, oval-shaped; iris bicolored, golden-orange dorsally, copper-red with thick black intervening ventrally; with wide black longitudinal stripe running medially, incorporating black pupil (Figure 3D).

**Color of holotype in preservative**: After preservation in ethanol for two years, coloration pattern of holotype resembles that observed in life; however, yellowish and reddish tints faded completely, turning beige-grey, brownish-black patterns on lateral surfaces appeared brownish-grey; ventral coloration lost purple and bluish tints and looked beige-grey with brown spots. 

**Variation**: All individuals in type series were very similar in morphology, body proportions, and body coloration; measurements of type series are shown in Table 4 and representative photographs showing variation in dorsal and ventral coloration of four male paratypes in life are given in Figure 5 and Figure 6. All specimens show certain variation in number and position of small dark-brown spots on dorsum (Figure 5A, Figure 6) and ventral pattern, including size of two oblique brownish spots in chest area (Figure 5B). Coloration of *Theloderma auratum* **sp. nov.** showed slight variation in response to diel period and microhabitat conditions. In life, coloration of dorsum was somewhat lighter nocturnally than during daytime, with dorsal surfaces looking light-beige to cream. Variation in color related to diel period, stress, and other conditions has been reported for other species of *Theloderma* (McLeod & Ahmad, 2007; Rowley et al., 2011).

**Tadpole description**: Description of larval morphology was based on five tadpoles (Gosner stages 29–36) (ZISP 13422–13426) (see Referred specimens for details). Identification of tadpoles was confirmed by 16S rRNA partial sequencing (see Table 2). The main morphometric parameters of the tadpoles are given in Table 5. Ontogenetic stages of *Theloderma auratum* **sp. nov.** are shown in Figure 7. Details of tadpole morphology are presented in Figure 8.

**External morphology**: Body oval, wider than high, body height 71%–78% of body width. Body longer than wide: body width 66%–74% of body length. Snout blunt, rounded. Tail more than two times longer than body, body length 55%–65% of tail length (Figure 7A, B); 23–28 myotomes discernable in lateral view. Nostrils rounded, oriented anterodorsally, located almost at same distance to snout as to eye (RN/NP ratio 71%–105%). Internarial distance (IND) 56%–77% of interorbital distance (IOD). Eyes with dorsal orientation. Eye diameter (ED) 9%–18% of body length (BL). Eye-nostril distance (END) larger than eye diameter (ED). Spiracle single, sinistral, located closer to posterior portion of body, directed laterally. Distance from tip of snout to spiracle opening 67%–75% times body length. Vent tube in medial position, with aperture located on same line as margin of ventral tail fin. Height of tail musculature at highest portion 58%–74% of tail height and 50%–57% of maximum body height. Maximum height of dorsal tail fin 34%–42% of maximum tail height. Ventral tail fin same height as dorsal tail fin; maximum height of lower tail fin 35%–46% of maximum tail height. Ventral and dorsal tail fins start roughly at level of vent. Dorsal and ventral tail fins slightly higher in posterior one third of tail length; tail tip gently rounded (Figure 7C; Figure 8A).

**Oral disk**: Mouth with ventral orientation (Figure 8C), oral disk wide, elliptical, width 38%–44% of body width. Lower labium fringed with double row of finger-shaped papillae (Figure 8D). Submarginal papillae located laterally of upper and lower labium. Number of marginal papillae in one row 80–85. LTRF: 5(3–5)/3. Number of keratodonts 24–28 on 1 mm. Lower sheath “V”-shaped; upper sheath “U”-shaped, both with distinctly serrated edges (Figure 8D).

**Coloration in life**: Body strongly pigmented: dorsally and ventrally uniformly dark-grey (Figure 7C, D). In dorsal view, eyes not totally visible on strongly pigmentated background (Figure 7C). Iris black. Spiracle slightly pigmented. Dorsal and ventral tail fins translucent at edges with greyish coloration, much lighter than on body. 

**Coloration in preservative**: In ethanol, all parts of tadpoles were less intensively pigmented and turned greyish. In preservation, myotomes of tail muscles were more discernable than in life.

**Figure 9:**
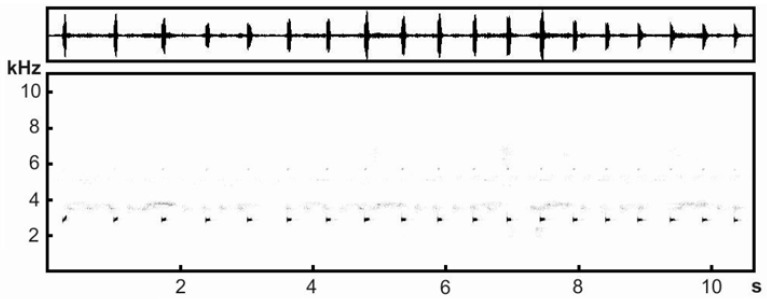
Oscillograms (top) and sonograms (bottom) of male advertisement calls of holotype of *Theloderma auratum* sp. nov. (ZMMU A-5828), recorded at 21.5 °C (Kon Chu Rang Nature Reserve, Gia Lai Province, Tay Nguyen Plateau, central Vietnam)

**Advertisement call**: Advertisement calls of *Theloderma auratum* **sp. nov.** were uttered in a series of 14.27±1.31 (7–21, *n*=15) tonal calls (Figure 9), resembling an orthopteran call to the human ear. Series duration varied from 3.04 s to 11.34 s (7.88±0.62 s, *n*=15) and the interval between successive series comprised 21.61±2.57 s (12.16–44.4 s, *n*=13). Call duration varied from 30 ms to 75 ms (61±0.5 ms, *n*=214) and inter-call interval varied from 411 ms to 842 ms (529±6.76 ms, *n*=199) usually decreasing gradually from the beginning to end of the series (Figure 9). Call repetition rate within the series was 1.68±0.03 calls/s (1.53–1.98 calls/s, *n*=15). Initial fundamental frequency was 2 777±3 Hz (2 570–2 900 Hz, *n*=214) and final fundamental frequency was 2 856±4 Hz (2 710–3 090 Hz, *n*=214). The initial and final fundamental frequencies also represented the minimum and maximum fundamental frequencies, respectively. Frequency modulation was usually expressed in the weak lift of fundamental frequency during the whole call. The presence of harmonics varied between/within recordings and mostly depended on recording quality (e.g., sensitivity of recording equipment, distance from vocalizing animal, signal volume, and background noise). Calls from the highest quality recording contained poorly visible second harmonics (Figure 9). The maximum amplitude frequency varied from 2 760 to 2 950 Hz (2 829±2 Hz, *n*=214) and coincided with the fundamental frequency.

**Position in mtDNA genealogy and sequence divergence**: According to our mtDNA data, *Theloderma auratum* **sp. nov.** belongs to the subgenus *Theloderma* s. str. (Poyarkov et al., 2015), and is reconstructed as a sister species of *Th. palliatum* (Figure 2, clade II-B). Uncorrected genetic *P*-distances between *Theloderma auratum* **sp. nov.** 12S rRNA and 16S rRNA sequences and all homologous sequences of congeners included in our analyses varied from 8.9% (with sister species *Th. palliatum*) to 17.9% (with *Th. laeve*) (see Table 3). This value is higher than that observed between several currently recognized species of *Theloderma* (Table 3). 

**Distribution and biogeography**: The known distributions of *Theloderma auratum* **sp. nov.** and its sister species *Th. palliatum* are shown in Figure 1. To date, the new species is known from montane evergreen tropical forests of Tay Nguyen Plateau in the central Annamite (Truong Son) Mountains, and has been recorded in Gia Lai, Kon Tum, and Thua Thien-Hue provinces. It is anticipated that *Theloderma auratum* **sp. nov.** also occurs in the adjacent montane forests of Tay Nguyen Plateau; in particular, records from Quang Nam Province of Vietnam and Xekong Province of Laos are anticipated.

**Figure 10:**
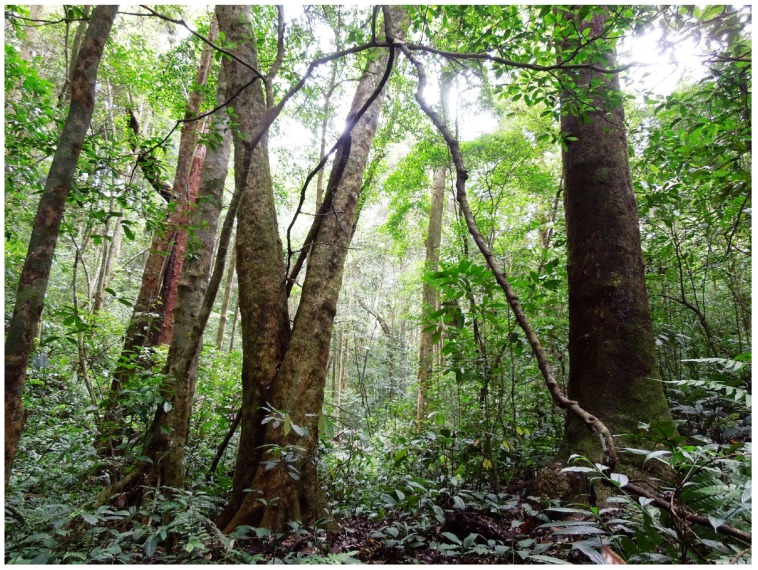
Habitat of *Theloderma auratum* sp. nov. at type locality; montane tropical forest in Kon Chu Rang Nature Reserve, Gia Lai Province, Tay Nguyen Plateau, central Vietnam (Photo by Alina V. Alexandrova)

**Natural history notes**: Our knowledge on the biology of *Theloderma auratum* **sp. nov.** is scarce. The new species was recorded in primary polydominant tropical montane evergreen forests of Tay Nguyen Plateau at elevations ranging from 800 to 1 400 m a.s.l.. Animals were recorded only in patches of primary undisturbed forest with complete multi-layered canopy and heavy undergrowth, suggesting the new species is a strict forest-dwelling specialist. At the type locality in Kon Chu Rang Nature Reserve (Gia Lai Province), the forest where the new species was recorded is dominated by large trees of the families Podocarpaceae (*Dacrydium elatum*, *Dacrycarpus imbricatus*), Magnoliaceae, Burseraceae (*Canarium* sp.), Myrtaceae (*Syzygium* sp.), Hamamelidaceae (*Simingtonia* sp.), Lauraceae (*Litsia* sp.), Rhodoliaceae (*Rhodolia* sp.), Fagaceae, Sterculiaceae (*Scaphium* sp.) (Figure 10).

*Theloderma auratum* **sp. nov.** is a very secretive species with apparently nocturnal activity. Animals were usually encountered at night (between 1900 h and 0300 h) after or during heavy rain; males were recorded when calling from shrubs or small trees ca. 20–90 cm above the ground. Diet of the new species remains unknown. In Kon Plong District, Kon Tum Province, both males and females were observed at an elevation of 1 000 m a.s.l. when sitting on leaves of shrubs at 40–50 cm from the ground; animals were active at an air temperature of 20–22°C and 100% humidity.

Other species of anurans recorded syntopically with the new species at the type locality included *Ingerophrynus galeatus* (Günther, 1864), *Kurixalus banaensis* (Bourret, 1939), *Rhacophorus annamensis* Smith, 1924, *Rhacophorus rhodopus* Liu & Hu, 1960, *Rh. robertingeri* Orlov, Poyarkov, Vassilieva, Ananjeva, Nguyen, Nguyen & Geissler, 2012, *Polypedates mutus* (Smith, 1940), *Rana johnsi* Smith, 1921, *Sylvirana nigrovittata* (Blyth, 1856), *Microhyla pulverata* Bain & Nguyen, 2004, and *Occidozyga vittata* (Andersson, 1942). In Kon Ka Kinh National Park (Gia Lai Province), the new species was recorded in sympatry with three other *Theloderma* species, including *Th. albopunctatum*, *Th. vietnamense*, and *Th. truongsonense*, with the latter species sharing the same biotopes as *Theloderma auratum* **sp. nov.** In Thac Nham Forest (Kon Plong Province), the new species was recorded in sympatry with *Th. albopunctatum*, *Th. gordoni*, and *Th. ryabovi*. 

**Reproductive biology**: Reproduction of the new species was observed under laboratory conditions. In terrarium, clutches of *Theloderma auratum* **sp. nov.** were found on the under-surface of snags (Figure 7) and were usually deposited 2–4 cm above the surface of the water. A clutch consisted of one or two, rarely three eggs, enveloped in slimy transparent external egg-capsules (see Figure 7A, B). Duration of embryonic development comprised 10–12 d at ambient air temperatures of 22–23°C. Tadpoles were fed on dry fish food and started active feeding 2–3 d after hatching. Duration of larval development from hatching to the end of metamorphosis was about 2.5 months. At metamorphosis, the main aspects of coloration and dorsal pattern corresponded to that of the adults, except the dorsal coloration pattern: metamorphs had a more intensive orange dorsum with orange reticulate pattern (Figure 7E), which disappeared with age. 

**Comparisons**: *Theloderma auratum* **sp. nov.** is easily distinguished from most species of the genus *Theloderma* by a combination of the following morphological attributes: (1) small to medium body size (SVL 21.8–26.4 mm); (2) absence of vomerine teeth; (3) smooth skin on dorsum with dorsal warts or asperities absent, (4) long obtuse snout with pointed snout tip, ESL/SVL ratio 16%–20%; (5) uniform golden-yellow dorsal coloration with weak golden-orange speckling and reticulations; (6) dark reddish-brown to black lateral stripes, separated from dorsal light coloration by sharp edge, forming very weak and shallow inguinal loop; and (7) bicolored iris, golden-orange dorsally, copper-red with thick black intervening ventrally, with a wide black longitudinal medial stripe.

The small body size, absence of vomerine teeth, and smooth dorsal skin lacking asperities or warts distinguishes *Theloderma auratum* **sp. nov.** from the large-sized *Theloderma* species: *Th. bicolor* (Bourret, 1937), *Th. corticale* (Boulenger, 1903), *Th. gordoni* Taylor, 1962, *Th. leporosum* Tschudi, 1838, *Th. moloch* (Annandale, 1912), and *Th. nagalandense* Orlov, Dutta, Ghate et Kent, 2006 (vs. large body size, large dorsal asperities, and vomerine teeth present).

Absence of hand webbing, reduced foot webbing, and completely smooth dorsum clearly distinguishes the new species from *Th. horridum* (Boulenger, 1903), *Th. stellatum* Taylor, 1962, *Th. vietnamense* Poyarkov, Orlov, Moiseeva, Pawangkhanant, Ruangsuwan, Vassilieva, Galoyan, Nguyen & Gogoleva, 2015, *Th. ryabovi* Orlov, Dutta, Ghate & Kent, 2006, *Th. phrynoderma* (Ahl, 1927), *Th. asperum* (Boulenger, 1886), and *Th. albopunctatum* (Liu & Hu, 1962) (vs. hand webbing well-developed in *Th. horridum*, rudimentary in remaining taxa; vs. foot webbing complete, dorsum with more or less developed warts and asperities in above-mentioned taxa).

The Malayan species *Th. licin* and *Th. asperum*, Indochinese species *Th. albopunctatum*, and poorly known *Th. baibungense* (Jiang, Fei & Huang, 2009) and *Th. pyaukkya* Dever, 2017 can be further distinguished from *Theloderma auratum* **sp. nov.** by whitish coloration of whole dorsum or large white blotches on dark dorsal background color (vs. golden-yellow dorsum with golden-orange reticulations and few dark spots in *Theloderma auratum* **sp. nov.**), greyish or whitish belly with brown reticulations (vs. whitish to bluish belly with small brown spots and blotches in *Theloderma auratum* **sp. nov.**), and uniform red coloration of iris (vs. distinctly bicolored golden/black iris in *Theloderma auratum* **sp. nov.**). Furthermore, *Th. licin* McLeod & Ahmad, 2007 from Sundaland and the Malayan Peninsula can be distinguished from the new species by the uniform dark-red coloration of iris (vs. distinctly bicolored golden/black iris in *Theloderma auratum* **sp. nov.**) and short rounded snout with ESL/HL ratio below 40% (vs. long pointed snout with ESL/HL ratio over 43% in *Theloderma auratum* **sp. nov.**).

*Theloderma annae* Nguyen, Pham, Nguyen, Ngo & Ziegler, 2016 can be easily differentiated from the new species by a golden-green iris with black reticulations and greenish-grey dorsal pattern (vs. distinctly bicolored golden/black iris and golden-yellow dorsum with golden-orange reticulations in *Theloderma auratum* **sp. nov.**) and presence of supernumerary palmar tubercles (vs. absent in *Theloderma auratum* **sp. nov.**). *Theloderma auratum* **sp. nov.** can be distinguished from two other small-sized *Theloderma* species found in northern Vietnam and southern China – *Th. rhododiscus* (Liu & Hu, 1962) and *Th. lateriticum* Bain, Nguyen & Doan, 2009 – by golden-yellow dorsum with golden-orange reticulations and few dark spots (vs. tea brown to dark grey dorsum in *Th. rhododiscus* and dorsum with deep brick-red background color with distinct black middorsal spot or blotches in *Th. lateriticum*), greyish belly or whitish belly with brown reticulations (vs. belly brownish-black scattered with grey-white network in both taxa), ventral surfaces of disks grey (vs. orange or red in *Th. rhododiscus*), completely smooth dorsum without asperities or warts (vs. distinctly white dorsal asperities present in both taxa), and distinctly bicolored golden/black iris (vs. uniformly dark reddish brown iris in both taxa). *Theloderma lacustrinum* Sivongxay, Davankham, Phimmachak, Phoumixay & Stuart, 2016 from Laos can be differentiated from the new species by its uniform red iris (vs. distinctly bicolored golden/black iris), beige-brown upper jaw (vs. uniformly dark reddish-brown upper jaw), and presence of large black blotches in inguinal region (vs. dark inguinal blotches absent).

*Theloderma petilum* (Stuart & Heatwole, 2004) can be easily differentiated from the new species by two chocolate-brown bands running from lateral surfaces of head toward groin, distinctly separated from brownish-beige dorsum and edged with white (vs. dark lateral bands black to reddish-brown, sharply contrasting with golden-orange dorsum with golden-orange reticulations in *Theloderma auratum* **sp. nov.**).

**Figure 11:**
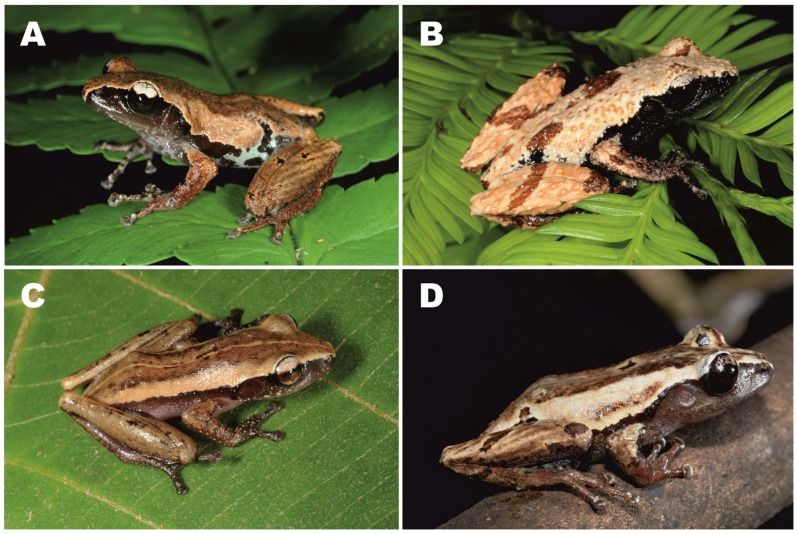
Small-bodied *Theloderma* species of central and southern Vietnam

Superficially, *Theloderma auratum* **sp. nov.** morphologically resembles other small-bodied *Theloderma* species found in central and southern Vietnam, namely *Th. laeve* (Smith, 1924), *Th. nebulosum* Rowley, Le, Hoang, Dau & Cao, 2011, *Th. truongsonense* (Orlov & Ho, 2005), and *Th. palliatum* Rowley, Le, Hoang, Dau & Cao, 2011. Thus, comparisons with these species appear to be the most pertinent: *Th. laeve* (Smith, 1924), *Th. nebulosum* Rowley, Le, Hoang, Dau & Cao, 2011, *Th. truongsonense* (Orlov & Ho, 2005), and *Th. palliatum* Rowley, Le, Hoang, Dau & Cao, 2011 (Figure 11). Table 6 summarizes the morphometric data on the small-bodied *Theloderma* species of central and southern Vietnam.

A: *Th. truongsonense* from Kon Ka Kinh N.P., Gia Lai Prov.; B: *Th. palliatum* from Chu Yang Sin N.P., Dak Lak Prov.; C: *Th. laeve* from Loc Bac Forestry, Lam Dong Prov.; D: *Th. nebulosum* from Ngoc Linh Mt., Kon Tum Prov. Photos A–C by Nikolay A. Poyarkov; Photo D by Nikolai L. Orlov.

**Table 6:** Morphometric differentiation of small-bodied *Theloderma* species of central and southern Vietnam, resembling *Theloderma auratum* sp. nov.

**Species**	**SVL**	**A-G**	**HW**	**HL**	**HD**	**UEW**	**IOD**	**ED**	**TD**	**ESL**	**IND**	**END**	**TED**	**NS**	**FLL**
***Th. palliatum***	**26.1±0.4**	**12.3±0.5**	**8.5±0.2**	**10.1±0.1**	**4.2±0.1**	**2.8±0.1**	**3.4±0.2**	**3.3±0.4**	**2.3±0.2**	**4.4±0.1**	**2.7±0.2**	**2.5±0.1**	**1.0±0.1**	**1.8±0.1**	**15.0±0.5**
*n*=4	(25.5–26.6)	(11.6–12.9)	(8.1–8.7)	(9.9–10.2)	(3.9–4.4)	(2.6–3.0)	(3.2–3.8)	(3.0–4.0)	(2.1–2.6)	(4.3–4.6)	(2.5–2.9)	(2.3–2.6)	(1.0–1.2)	(1.5–1.9)	(14.2–15.6)
***Th. truongsonense***	**24.8±0.8**	**11.2±0.7**	**8.1±0.3**	**9.2±0.2**	**3.5±0.3**	**2.0±0.1**	**3.0±0.1**	**3.2±0.0**	**1.8±0.1**	**3.8±0.1**	**2.0±0.1**	**2.3±0.1**	**0.7±0.0**	**1.3±0.1**	**13.7±0.2**
*n*=5	(23.6–26.4)	(10.3–12.7)	(7.8–8.8)	(9.0–9.6)	(3.2–3.8)	(1.8–2.2)	(2.9–3.1)	(3.2–3.2)	(1.7–1.9)	(3.5–4.1)	(1.9–2.0)	(2.2–2.4)	(0.7–0.7)	(1.2–1.3)	(13.3–14.1)
***Th. laeve***	**25.5±1.6**	**12.0±0.4**	**7.8±0.3**	**8.6±0.4**	**3.0±0.2**	**1.7±0.0**	**2.5±0.1**	**3.2±0.2**	**1.6±0.0**	**3.9±0.2**	**2.0±0.1**	**2.4±0.2**	**0.8±0.1**	**1.6±0.1**	**13.7±0.5**
*n*=5	(23.0–28.7)	(11.4–12.9)	(7.4–8.2)	(7.9–9.5)	(2.8–3.4)	(1.6–1.8)	(2.3–2.7)	(2.8–3.4)	(1.6–1.6)	(3.6–4.0)	(1.8–2.2)	(2.1–2.7)	(0.6–0.9)	(1.4–1.7)	(13.0–14.5)
***Th. auratum* sp. nov.**	**25.3±1.0**	**12.0±0.4**	**7.9±0.2**	**9.8±0.4**	**4.1±0.2**	**2.2±0.1**	**3.0±0.1**	**3.3±0.1**	**1.8±0.1**	**4.4±0.2**	**2.3±0.1**	**2.8±0.1**	**0.7±0.0**	**1.7±0.1**	**13.9±0.9**
*n*=7	(21.8–26.4)	(11.2–12.8)	(7.5–8.2)	(9.2–10.4)	(3.6–4.4)	(2.0–2.5)	(2.8–3.2)	(3.2–3.5)	(1.7–2.0)	(4.2–4.7)	(2.1–2.4)	(2.7–3.0)	(0.6–0.8)	(1.5–2.0)	(12.4–15.5)
	**LAL**	**ML**	**FFL**	**TFL**	**FTD**	**NPL**	**MCTe**	**HLL**	**FL**	**TL**	**FOT**	**FTL**	**FFTL**	**HTD**	**MTTi**
***Th. palliatum***	**5.7±0.4**	**7.3±0.3**	**2.2±0.0**	**4.8±0.5**	**1.3±0.2**	**2.2±0.1**	**1.5±0.5**	**41.9±0.7**	**12.2±0.6**	**14.3±0.6**	**11.0±0.1**	**2.1±0.0**	**5.7±0.6**	**0.9±0.1**	**1.1±0.1**
*n*=4	(4.9–6.0)	(6.9–7.7)	(2.1–2.3)	(4.3–5.8)	(1.1–1.6)	(2.1–2.4)	(1.1–2.4)	(41.2–43.0)	(11.5–13.5)	(13.7–15.5)	(10.9–11.2)	(2.1–2.2)	(4.8–6.6)	(0.7–1.0)	(1.0–1.2)
***Th. truongsonense***	**5.0±0.1**	**6.4±0.4**	**2.0±0.1**	**4.1±0.5**	**1.2±0.1**	**1.4±0.1**	**0.8±0.1**	**39.3±2.5**	**11.3±0.4**	**12.5±0.4**	**9.5±0.1**	**1.5±0.1**	**5.3±0.1**	**0.9±0.1**	**1.0±0.0**
*n*=5	(5.0–5.2)	(6.0–6.9)	(1.8–2.2)	(3.3–4.7)	(1.1–1.3)	(1.2–1.6)	(0.7–0.9)	(35.6–42.8)	(10.5–11.9)	(11.7–13.0)	(9.3–9.6)	(1.4–1.7)	(5.1–5.5)	(0.8–1.0)	(1.0–1.0)
***Th. laeve***	**5.1±0.2**	**6.1±0.2**	**1.9±0.1**	**4.2±0.2**	**1.1±0.1**	**1.1±0.1**	**0.5±0.1**	**40.5±1.7**	**11.7±0.6**	**13.4±0.5**	**10.4±0.3**	**2.0±0.1**	**4.7±0.3**	**1.2±0.0**	**0.9±0.2**
*n*=5	(4.8–5.4)	(5.8–6.4)	(1.7–2.0)	(3.6–4.7)	(1.0–1.3)	(1.0–1.2)	(0.3–0.7)	(37.6–43.0)	(10.1–13.3)	(12.3–14.2)	(9.9–10.9)	(1.9–2.2)	(4.3–5.3)	(1.2–1.3)	(0.6–1.1)
***Th. auratum* sp. nov.**	**5.4±0.4**	**6.8±0.3**	**2.2±0.1**	**4.7±0.2**	**1.2±0.2**	**1.5±0.3**	**0.7±0.1**	**39.8±1.2**	**11.9±0.4**	**13.8±0.5**	**10.8±0.3**	**1.5±0.1**	**5.4±0.2**	**1.0±0.2**	**1.1±0.1**
*n*=7	(4.8–5.8)	(6.2–7.5)	(2.0–2.4)	(4.3–5.1)	(0.8–1.4)	(1.1–1.9)	(0.6–0.8)	(36.1–42.0)	(11.0–12.4)	(12.5–14.5)	(10.4–11.5)	(1.3–1.7)	(5.0–5.9)	(0.6–1.3)	(1.0–1.3)

all data in mm, for males only; mean±*SD* and Max.-Min. values are given. For abbreviations, see “Materials and methods”.

*Theloderma nebulosum* (Figure 11D) can be distinguished from the new species by small sparse dorsal asperities present (vs. absent), brown dorsum with darker patterning (vs. golden-yellow dorsum with golden-orange reticulations and few dark spots), and dark brown to black ventral surfaces with bluish marbling (vs. whitish to bluish belly with small brown spots and blotches).

*Theloderma truongsonense* (Figure 11A) from the central and southern Annamite (Truong Son) Mountains can be distinguished from *Theloderma auratum* **sp. nov.** by the following morphological attributes: dorsal skin shagreened with numerous small asperities scattered on dorsum and dorsal surfaces of head including upper eyelids (vs. completely smooth dorsum with only few (3–5) flat superciliary tubercles), comparatively shorter snout with rounded snout tip, ESL/SVL ratio below 16% (vs. tapering snout with pointed tip, comparatively longer, ESL/SVL ratio above 17%), dorsum brown with darker patterning (vs. golden-yellow dorsum with golden-orange reticulations and few dark spots), dorsal edge of dark lateral stripe irregular, deep inguinal loop or large black inguinal blotch surrounded by bluish to turquoise edging always present (vs. dark lateral stripe with smooth sharp edge, inguinal loop absent or shallow), comparatively wider and shorter head, HW/HL ratio above 85% (vs. comparatively narrower and longer head, HW/HL ratio below 84%), and comparatively shorter tibia, TL/SVL ratio below 52% (vs. comparatively longer tibia, TL/SVL ratio above 53%).

*Theloderma laeve* (Figure 11C) from low to mid-elevations of Langbian Plateau, southern Vietnam, can be diagnosed from *Theloderma auratum* **sp. nov.** by the following combination of morphological characters: dorsum beige with brownish patterning and thin light middorsal stripe (vs. golden-yellow dorsum with golden-orange reticulations and few dark spots), lateral stripe brownish-violet (vs. black to dark brown lateral stripe), ventral surfaces uniform violet-grey with no dark patterning (vs. whitish to bluish belly with small brown spots and blotches), head short, HL/SVL ratio below 35% (vs. head comparatively longer, HL/SVL ratio over 37%), head width slightly less than head length, HW/HL ratio 86%–93% (vs. head notably longer than wide, HW/HL ratio 78%–84%), comparatively shorter snout with rounded snout tip, ESL/SVL ratio 14%–16% (vs. tapering snout with pointed tip, comparatively longer, ESL/SVL ratio 17%–20%), and comparatively smaller interorbital distance, IOD/SVL ratio 9%–10% (vs. IOD/SVL ratio 11%–14%).

From its sister species, *Th. palliatum* (Figure 11B), which inhabits high elevations of the Langbian Plateau, *Theloderma auratum* **sp. nov.** can be distinguished by the following morphological features: completely smooth dorsum (vs. notably tuberculated dorsum, with dorsal surfaces of body, head, and limbs covered with many tubercles and asperities), golden-yellow dorsum with golden-orange reticulations and few dark spots (vs. beige dorsum with large light-brown blotches on upper eyelids, scapular, and sacral area), dark lateral stripe with smooth sharp edge, inguinal loop shallow or absent, large black inguinal blotch absent (vs. dorsal edge of dark lateral stripe irregular, large black inguinal blotch or dark inguinal loop present), whitish to bluish belly with small brown spots and blotches (vs. almost black belly with thin bluish reticulations), snout dorsally pointed (vs. snout dorsally obtusely rounded), comparatively smaller tympanum, TD/SVL ratio 7%–8% (vs. comparatively larger tympanum, TD/SVL ratio 9%–10%), and comparatively larger eye to nostril distance, END/SVL ratio 11%–13% (END/SVL ratio 9%–10%).

Tadpoles of *Theloderma auratum* **sp. nov.** superficially resemble the tadpoles of *Th. nebulosum* described by Rowley et al. (2011); however, they can be distinguished from the latter by LTRF 5(3–5)/3 (vs. LTRF 4(2–4)/3 in *Th. nebulosum*). Tadpoles of *Th. laeve* and *Th. palliatum* also exhibit LTRF 4(2–4)/3 (see Orlov et al., 2012). 

**Etymology**: The specific name “*auratum*” is a Latin adjective in the nominative singular (neutral gender), derived from Latin “*aurum*” for “gold”, referring to the golden-yellowish dorsal coloration of the new species. 

**Recommended vernacular name**: We recommend the following common name in English: Golden Bug-Eyed Frog. Recommended vernacular name in Vietnamese: Ếch Cây Sần Vàng.

**Conservation status**: *Theloderma auratum* **sp. nov.** is, to date, known from five localities in the Tay Nguyen Plateau of central Vietnam. Further research is required to estimate its actual distribution, population trends, and possible threats. It appears that the new species is associated with primary undisturbed montane forests and may be affected by growing anthropogenic pressure and forest destruction, as observed in different areas of central Vietnam. Given the available information, we suggest *Theloderma auratum* **sp. nov.** be tentatively considered as a Data Deficient species following IUCN’s Red List categories (IUCN, 2001).

## DISCUSSION

Our phylogenetic data largely confirmed the phylogeny of *Theloderma* as given by Poyarkov et al. (2015), Nguyen et al. (2015), and Sivongxay et al. (2016), but included several taxa not analyzed in these studies. Our data supported subdivision of *Theloderma* into two major clades, corresponding to the subgenera *Stelladerma* (Clade I) and *Theloderma* s. str. (Clade II). Furthermore, *Th. annae* was reconstructed as a sister species of *Th. nebulosum* with high node support, and *Th. moloch*, *Th. phrynoderma*, and *Th. ryabovi* were supported as a monophyletic sister group to the *Th. asperum* complex.

Our research clearly demonstrated that current understanding of the diversity of *Theloderma* is far from complete. Within *Th. asperum*, our data strongly suggested paraphyly of *Th. pyaukkya* from Myanmar with respect to *Th. baibungense* from the eastern Himalayas. In addition, *Th. pyaukkya* could be subdivided into two highly divergent non-monophyletic lineages from northern and central Myanmar (*P*-distance 3.9%). Dever (2017) described *Th. pyaukkya* without including *Th. baibungense* in phylogenetic analysis and without examination of *Th. baibungense* specimens; comparisons of *Th. pyaukkya* in this paper do not allow the discrimination of *Th. pyaukkya* from *Th. baibungense*. Our data indicated that the taxonomic status of *Th. pyaukkya* lineages requires careful reconsideration: synonymy of this species with *Th. baibungense* can be assumed; however, high genetic differentiation between its lineages suggests that taxonomic reassessment of this group is necessary. Other examples of high intraspecific genetic distances included Indochinese species *Th. lateriticum* (*P*=3.6%), *Th. truongsonense* (*P*=3.8%), and *Th. albopunctatum* (*P*=2.5%); these results suggest the possible presence of cryptic species in these complexes and incomplete taxonomy of the group. Finally, *Theloderma* sp. from Gia Lai Province, previously identified as *Th. laeve* by Nguyen et al. (2015), clearly represented a deeply divergent mtDNA lineage sister to *Th. laeve* s. str. (*P*=9.0%) and likely corresponds to a yet undescribed species.

Our study confirmed significant species richness underestimation in *Theloderma* and reported on a new species of *Theloderma* from the Tay Nguyen Plateau of the Annamite (Truong Son) Mountains in central Vietnam. This mountainous area harbours the highest diversity of amphibian species in Indochina (Geissler et al., 2015) and is recognized as a local center of herpetofaunal diversity with many new species of amphibians and reptiles described from the plateau in the last decade (Poyarkov et al., 2014, 2017; Rowley et al., 2010, 2011, 2014, 2016; Nguyen et al., 2018, Nguyen et al., in press). Our data indicate that knowledge on amphibian diversity of this area is still far from complete and further diversity is likely to be revealed with additional survey efforts. Habitat loss and modification is a continued threat in the region (Meyfroidt & Lambin, 2008), and is widely recognized as the greatest threat to amphibians in Southeast Asia. Range-restricted species are at greatest risk, and include *Theloderma auratum* **sp. nov.**, which appears to be a forest specialist restricted to relatively undisturbed broadleaf evergreen montane forests. Further survey efforts and additional taxonomic studies to understand the true diversity of amphibians in the region are urgently required for effective conservation management.

## Appendix I

**List of examined specimens.**

*Theloderma laeve*: **ZMMU A-4569** (Bu Gia Map N.P., Binh Phuoc Province, southern Vietnam; 1 male, holotype of *Th. bambusicolum*); **ZMMU A-4570** (Bu Gia Map N.P., Binh Phuoc Province, southern Vietnam; 2 males, paratypes of *Th. bambusicolum*); **ZMMU A-4573** (Bu Gia Map N.P., Binh Phuoc Province, southern Vietnam; 3 females, paratypes of *Th. bambusicolum*); **ZMMU A-4571** (Cat Loc area, Cat Tien N.P., Lam Dong Province, southern Vietnam; 4 males, 1 female, paratypes of *Th. bambusicolum*);

*Theloderma palliatum*: **ZMMU A-5835** (Chu Yang Sin N.P., Dak Lak Province, southern Vietnam; 4 males);

*Theloderma truongsonense*: **ZMMU A-5827** (SaoLa Reserve, ARoang area, ALuoi district, Thua-Thien - Hue Province, central Vietnam; 5 males);

*Theloderma auratum* **sp. nov.**: **ZMMU A-5828** (Kon Chu Rang N.R., Gia Lai Province, central Vietnam; 1 male, holotype); **ZMMU A-5829** (Kon Chu Rang N.R., Gia Lai Province, central Vietnam; 1 male, paratype); **ZMMU A-5830** (Kon Chu Rang N.R., Gia Lai Province, central Vietnam; 1 male, paratype); **ZMMU A-5831** (Kon Ka Kinh N.P., Gia Lai Province, central Vietnam; 1 male, paratype); **ZMMU A-5832** (Thac Nham Forest, Kon Plong area, Kon Tum Province, central Vietnam; 1 male, paratype); **ZMMU A-5833–A-5834** (SaoLa Reserve, ARoang area, ALuoi district, Thua-Thien - Hue Province, central Vietnam; 2 males).
